# Green city development principles (GCDP) implementation level and its effects on lagos megacity residents’ well-being, Nigeria: A qualitative study

**DOI:** 10.1371/journal.pone.0354733

**Published:** 2026-08-04

**Authors:** Eghosa N. Ekhaese, Kingsley O. Dimuna, Aniefon H. Essien

**Affiliations:** 1 Department of Architecture, Covenant University, Ota, Ogun State, Nigeria; 2 Department of Architecture, Ambrose Alli University, Ekpoma, Nigeria; Federal University Otuoke, NIGERIA

## Abstract

Cities are subject to rapid urbanisation, climate change and environmental challenges. Lagos, being the 4th most populous city in the world and Africa, is also facing these challenges. The study investigates the impact of implementing the green city development principle (GCDP) as a framework to enhance residents’ well-being in Lagos, Nigeria. The instruments used were an In-Depth Interview (IDI) guide, an observation schedule and a GCDP checklist to determine the Lagos Megacity implementation level. The interpretivist and constructivist paradigms were engaged as the research philosophy. The findings present several obstacles that hinder GCDP’s practical implementation and the relationship between GCDP implementation and residents’ well-being in a megacity. Based on the findings, the study provides recommendations for policymakers and urban planners to enhance the effectiveness of GCDP implementation and promote sustainable urban development in Lagos while endorsing GCDP initiatives to improve well-being in cities.

## Introduction

Environmental and climate-related challenges in urban policies are concerns facing communities today [1]. Traffic and overcrowding, poor air quality, greenhouse gas (GHG) emissions, excessive noise, urban sprawl, low-quality built environment, and wastewater and waste treatment are all issues that most cities face [2]. The growing resource use by urban residents contributes significantly to these consequences resulting from changes in urban lifestyles and is linked to poverty and other socio-economic factors [3]. People were concerned about pollution from atomic testing, millions of cars and companies’ emissions, the devastation of rivers and lakes, and the elimination of farmland/woodland due to suburban construction [4]. Recent research highlights that harmful products, such as pesticides and herbicides, are causing population declines in birds, insects, and other animals in our ecosystem [5]. The effects of industrialisation and rapid urbanisation on the planetary systems have become apparent [6]. According to [7], the world’s urban population will reach 66 per cent by 2050, underscoring the prominence of cities. The expanding population due to urban development has heightened discussion of city sustainability, as cities are resource-intensive, accounting for 75% of carbon emissions and energy consumption [8]. Cities are responsible for some global GHG emissions. Cities experience the effects of climate change, with poor populations being the most vulnerable [9]. As a result, many cities are rethinking their development strategies by designing for a green and resilient future, protecting the environment, and devising context-specific solutions [10,11]. The introduction of green development has helped to preserve resources and the natural environment through green buildings and cities [12]. The green movement is sustainable [13]. Today, the green movement addresses environmental challenges such as global warming and climate change, fisheries depletion, nuclear proliferation, wetlands protection, species extinction and other issues [14]. However, urbanisation is happening at an alarming rate in Lagos, Nigeria. According to [15], Lagos is one of three megacities in Africa, and its projected annual population growth rate is 2.5% or higher, adding approximately 730,000 people per year.

Against this background, the investigators selected Lagos, Nigeria, as a case study because of the city’s rapid urbanisation as a megacity. The study aims to investigate the impact of implementing the GCDP framework on residents’ well-being in Lagos, Nigeria, and to promote residents’ quality of life (QoL). Using the following research objectives: identify the obstacles to GCDP implementation in Lagos, Nigeria, ascertain the GCDP implementation level in Lagos Megacity, Nigeria and assess the effect of GCDP implementation on residents’ well-being in Lagos Megacity, Nigeria. The theoretical significance of the GCDP implementation in Lagos lies in expanding the body of knowledge on sustainable and resilient urban development amid rapid urbanisation, particularly in African megacities. It further advances urban sustainability theory, urbanisation challenges, and the complex Interplay of Factors. It informs the development of new theoretical frameworks for analysing and addressing the challenges of urban development in similar contexts globally. The practical significance lies in providing actionable insights and recommendations for stakeholders to inform policy and planning, guide resource allocation, promote sustainable urban development practices, improve residents’ well-being, and create a more sustainable and resilient Lagos. The scope encompasses the implementation of GCDP, residents’ well-being, the Lagos Megacity Context, and an exploration of the successes, challenges, and potential areas for improvement in the implementation. The limitations stem from the complexities of urbanisation, the data collection challenges in such a dynamic environment as Lagos’ megacity, generalizability, time sensitivity, and potential for bias. The following section systematically and methodically investigates the existing literature to situate the subject matter at the forefront of research properly.

## Literature reviews

The author conducted searches using several databases, including Google Scholar, ResearchGate, ScienceDirect, and Scopus, using the stated keywords and grouping them. Other websites, perhaps the World Health Organisation library databases, were employed to search for data. The search process involved several steps: identifying significant terms related to the research question (based on PRISMA), listing keywords from existing articles relevant to our study, searching for synonyms and alternative terms, using Boolean operators (OR and AND) to connect significant terms and employing an asterisk (“*”) as a wildcard operator. The keywords employed were within the vocabulary commonly used for this topic, including “Green City Development Principles” AND “Green City Development Principles Implementation” AND “Health” OR “Well-being” AND “Green Urbanism”. To select suitable studies, the reviewers searched articles and vetted them based on titles, abstracts, and full text. The literature review revealed that most research findings were in the Global North. However, only a few were in the Global South, and the analysis focused on the built environment; some studies examined specific communities, such as African neighbourhoods in Botswana. Our inclusion criteria focused on empirical studies and literature reviews examining the effects of implementing Green City Development Principles on residents’ well-being. The authors explicitly considered works published within the last ten years. The reviewers excluded articles that did not address these issues, lacked empirical evidence to support their claims, and were not written in English or published in peer-reviewed journals more than ten years ago.

### Green city development (GCD) and Its benefits

According to [16], green city development is a concept of property growth that reflects the environmental and social consequences of building. The sub-categories are community/cultural sensitivity, Environmental responsiveness and resource efficiency [17]. Environmental receptiveness acknowledges nature’s fundamental value while minimising ecological damage. Resource efficiency involves conserving energy and ecosystems by using fewer resources. Community/cultural sensitivity identify the particular cultural values that each society holds and incorporates them into property development [18]. As part of GCD, numerous core themes such as energy efficiency, sustainable and low-carbon transportation systems, waste reduction and management, water cycle management, green and resilient infrastructure, increased green areas, and integrated planning help develop [19]. Adopting a green development model in cities fosters benefits for both inhabitants and the natural environment. According to [20], the ultimate goal of GCD is to ensure urban environments adapt effectively to location, climate, orientation and context. Using natural assets, such as sunshine and wind movement, is soft(er), cleaner, and more efficient, provides a favourable microclimate, and produces no CO2 emissions. However, energy producers fueled by renewable energy sources minimise waste. It is based on a closed-loop ecology with extensive recycling, reuse, remanufacturing, and composting [21].

According to [22], GCD encourages intra-city roads, conservation of previously urbanised areas, reduced use of automotive and public transportation, walking, the generation and use of renewable energy, and a lower per capita cost of infrastructure supply. Furthermore, GCD promotes natural resource management and increases community engagement/decision-making [23]. GCD prevents negative aspects such as traffic congestion, urban sprawl, poor public services, and informal settlements [24]. According to [25], GCD minimises disaster risk, improves air and water quality, promotes resident connection to the natural environment, improves well-being and biodiversity, offers serene city centres and other public areas and enhances aesthetic amenities. Existing research shows a link between GCD and climate change mitigation [26].

### Green urbanism and its principles

According to [27], green urbanism is the development of communities that benefit humans and the environment. It is an endeavour to design greener locations, neighbourhoods and lifestyles while using less of the Earth’s resources. Green urbanism requires the participation of architects, landscape architects, engineers, urban designers and planners, ecologists, psychologists, sociologists, economists, physicists, transportation planners, and other specialists [28]. Green urbanism minimises the service elements at each stage of the city life cycle, including their extraction, fabrication, assembly and transportation into the buildings [29]. Also, their recycling eases and value when the building’s life is complete. According to [30], the concept is to identify priorities and develop intelligent means of caring for and sustaining the Earth. The effort aims to produce positive, lasting changes that improve the environment. Green urbanism is a theory that comprises several principles [31]. According to [32], 17 pragmatic and integrative principles of green urbanism cover all areas of sustainable development and support best-practice models. These include: 1. Green, resilient infrastructure, 2. Renewable Energy for Zero CO2 Emissions, 3. Climate and Context, 4. Water, 5. Landscape, Gardens and Biodiversity, 6. Intelligent Systems and Smart Cities, 7. Local and sustainable materials with less embodied energy, 8. Density and retrofitting of existing districts, 9. Green buildings and neighbourhoods, 10. using passive design principles, 11. Livability, Healthy Communities and Mixed-Use Programmes, 12. Urban governance, leadership, and best practices, 13. Cultural heritage, identity, and sense of place, 14. Local food and short supply chains, 15. Education, research, knowledge, and Strategies for cities in developing countries, 16. Zero Waste City and 17. Sustainable transport and good public space [33].

### GCDP implementation

According to [34], this principle calls for construction using local and regionally sourced materials with less embodied energy. The aim is to shorten the supply chain for the building sector and focus on low-environment-impacting and recycled materials, as well as waste reduction. The guiding principles of GCDP are to minimise non-renewable energy consumption and waste, use environmentally preferable products, protect and conserve water, and improve indoor air quality [35–37]. Best described as a city-focused sustainability movement, the emerging GCDP encompasses thousands of urban areas worldwide striving to reduce their environmental impacts by reducing waste, expanding recycling, lowering emissions, and increasing housing density [38]. A Green City Action Plan (GCAP) will require a systematic approach to addressing its urban environmental challenges with tailored actions and visions [39]. A green city is an urban enclave whose design, construction, and operation prioritise the preservation of the natural world alongside the inhabitants’ economic, social, and physical health and wellness [40]. Therefore, to implement GCDP and develop a green city project, there are six ways to ensure a greener future, including access to public resources, transportation, green public spaces, green architecture, water conservation, and waste management [41].

### Global case studies

#### Best green policy initiatives and practices.

Cities in the Global North and Global South are implementing GCDPs in all aspects of urban planning. Examples of global case studies include *Copenhagen, Denmark; Stockholm, Sweden; Vancouver, Canada; Berlin, Germany; and The Garden City: Singapore.*

*Copenhagen, Denmark –* Cited as a green city, experts confirm it as the world’s greenest metropolis since 2017, when the C40 group of cities acknowledged its urban ecosystem. Copenhagen has pioneered a green economy and connected partnerships in its approach to eco-innovation, collaborating with companies, universities, and civil society to build forums that foster green development and create jobs. The European Green Capital Award committee named Copenhagen the European Green Capital in 2014. Copenhagen will become the world’s first carbon-neutral city by 2025. Since adopting the first-ever Climate Plan in 2009, the city has already achieved reductions in carbon emissions. The city’s environmental policy leadership includes district heating, renewable energy, waste management, the cleanup of the old industrial harbour, and the encouragement of cycling [42,43]. It plans to reduce losses to 6 per cent by 2025 by using water metering to reduce waste [44,45]. Statistics from 2013 showed that 96 per cent of Copenhagen residents lived within a 15-minute walk of a green or blue area. Plans are in place to further improve access to recreational areas. There are investments to make Copenhagen’s old harbour safe for swimming and fishing. Copenhagen established two city green initiatives, a school garden project and launched 22 local green partnership projects in 2011. The city planted 3600 trees, of which 6.03 per cent were adopted by local people, companies, or institutions [46].

*Stockholm, Sweden* **–** Stockholm is Sweden’s cultural, media, political and economic centre. The goal of being the world’s green capital is part of the City’s Vision 2030, which aims to make Stockholm world-class [47]. Stockholm was the European Green Capital in 2010 [48]. Stockholm, Sweden, is energy-efficient in the building sector, with over 50 years of district heating infrastructure [49,50]. Today, the city uses renewable fuel from waste or residual heat as 80% of the energy for district heating [51]. Its stringent national building regulations enhance the energy efficiency of new buildings. The goal is for all public transit to be fossil-fuel-free by 2025 [52]. The city is also prioritising pedestrians and cyclists in their urban planning.

Vancouver, Canada – according to [53], Vancouver is a significant city in western Canada, the mainland area of British Columbia. Vancouver City Council will obtain 100 per cent renewable energy (100% renewable) by 2050, a potentially imminent goal, given the city’s current hydroelectricity supply of over 95 per cent, which is renewable [54]. Around 90 per cent live within a 5-minute walk of green spaces, with an urban forest density of 18 per cent. The city protects urban green areas, parks, and street trees. Vancouver’s objective is to create energy-efficient buildings and their retrofits. All facilities in the city are to be carbon-neutral by 2030.

Berlin, Germany – according to [55], Berlin, the capital and primary urban centre, stands amid the North German plain. It is at an east-west commercial and geographic axis, as the capital of the Kingdom of Prussia since 1871 and of a united Germany. Germany’s capital is renowned as the city of green trends and is now a sustainable congress metropolis. Over the last decade, the Berlin-Brandenburg metropolitan region has embarked on a renewable energy and electric mobility agenda. The programme is part of the German Federal Government’s National Development Plan for Electric Mobility [56]. Berlin consists of 2,500 parks/gardens and 440,000 trees, making the Berlin city area 30% green space and woodland, with 180 km of navigable waterways.

The Garden City: Singapore [57] ranks at the top of the Asian Green City Index, with consistency across all categories. Singapore’s remarkable environmental performance since 1965 emphasises sustainability through all-inclusive planning, green space conservation and high-density development. Garden City has innovative water recycling plants, waste-to-energy facilities, and investments in transportation systems. The city is the finest in the waste category, generating only 307 kg of waste per person per year. Singapore is also at the top of the water category. Singapore has commissioned five (5) pioneering water-reclamation plants that treat wastewater using microfiltration, reverse osmosis, and ultraviolet technology. It can provide one-fifth of the city’s water supply.

## Materials and methods

**Fig 1:**
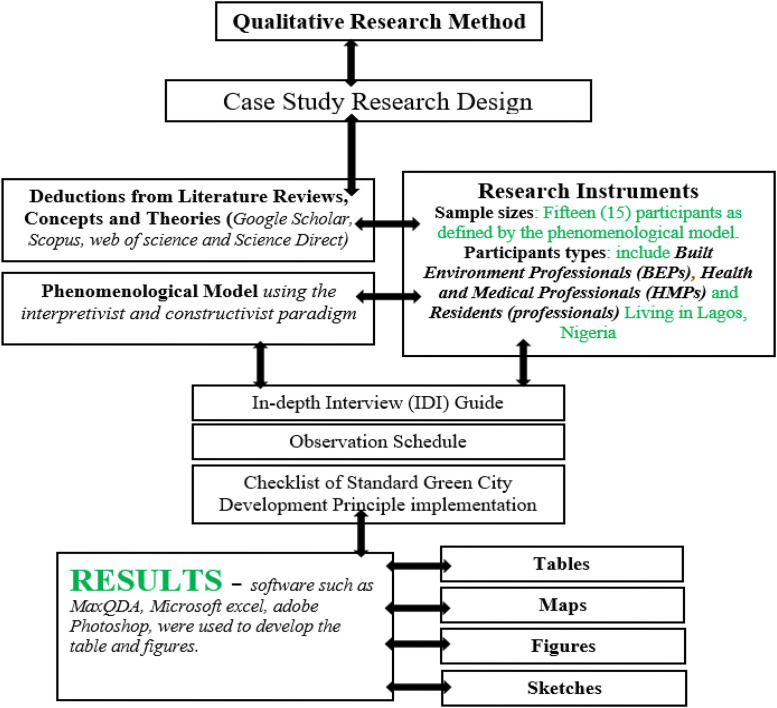
Structural flow of the research method/approach.

The research design is a case study examining the extent of GCDP implementation and its effects on residents’ well-being in the Lagos megacity, Nigeria. The case study design is suitable because it unfolds an existing situation. According to [58], the purpose is to examine the centre of interest in a specific area, feature, or unit of analysis, in this case, a city location, based on recognised standards. The study employed a qualitative research method using a positivist paradigm. The qualitative approach generally engages with text and language to derive research findings through firsthand experience, truthful reporting, and direct quotations from actual conversations. The research employed internet-based data and conducted textual analysis of relevant papers, journals, and publications on the subject. The investigators reported data to define a framework for evaluating the level of GCDP implementation and its effect on residents’ well-being, as shown in Fig 1. The literature sources were from Google Scholar, Scopus and ScienceDirect. The literature review identifies available GCDPs as a checklist for determining the degree of implementation of the principles in the Lagos megacity, Nigeria. The study used a phenomenological model to determine the size or number of participants/interviewees. Therefore, the research assistants and I conducted an in-depth interview with fifteen (15) participants. These participants include experts and professionals in Built Environments Professionals (BEPs), such as therapeutic architects, environmentalists, clinical psychologists and residents. Data collection processes took place between April 31 and September 30, 2023. Hence, the authors used language and data collection instruments based on direct observation and recorded in-depth interviews with respondents in the case study. The investigator turned some of the acquired data into figures, tables, or charts.

### Recruitment process

Participants for the study were 15 experts or professionals: Built Environment Professionals-BEPs (BEPs), health and medical professionals-HMPs and residents. The investigators conducted an in-depth individual interview (IDI) for 60–90 minutes. The investigators distributed 15 signed consent forms to participants in the study who expressed interest in the interview exercise. Following qualitative research recommendations to ensure appropriate representation of the lived experiences and diversity of the experts and residents, the investigators used purposive sampling based on six criteria: age, gender, marital status, education, occupation, and years of experience, as shown in Table 1.

**Table 1:** Demographic information of the participants.

S/N	Name	Age	Sex	Marital Status	Education	Occupation	Years of Experience / Length of Stay in Lagos
1	IDI 1	≥45	M	Married	M.Sc.	Environmentalist	25
2	IDI 2	≥45	M	Married	PhD	Residents (Community leader)	20
3	IDI 3	≥45	M	Married	PhD	Therapeutic Architect	18
4	IDI 4	≥45	M	Married	M.PH	Public health analysis	19
5	IDI 5	≥45	F	Married	PhD	Biomedical Chemist	16
6	IDI 6	≥45	F	Married	MD	Clinical Psychologist	15
7	IDI 7	≥45	F	Married	PhD	Environmental Biologist	21
8	IDI 8	≤45	M	Married	M.Sc.	Therapeutic Architect	17
9	IDI 9	≤45	M	Married	M.Sc.	Residents (urban planner)	15
10	IDI 10	≤45	M	Married	PhD	Residents	13
11	IDI 11	≤45	M	Married	M.Sc.	Residents (Community leader)	16
12	IDI 12	≤45	F	Married	PhD	Salutogenic Architect	14
13	IDI 13	≤45	F	Married	M.Sc.	Clinical Psychologist	12
14	IDI 14	≤45	M	Married	M.PH.	Public health analysis	11
15	IDI 15	≥45	F	Married	PhD	Clinical Psychologist	22

Mean Age: 46.3, Mean Years of E: 15.0

PhD- Doctor of Philosophy, M.PH- Master of Public Health, M.Sc- Master of Science, M- Male, F- Female.

### Sampling technique

The sampling techniques employed for the study are purposive, snowball and convenience sampling. These involve selecting participants based on specific criteria relevant to the research objectives [59], such as their knowledge and experience with green city initiatives or role in the community. Snowball sampling was used during the interview to recruit initial participants who would refer the researcher to other potential participants. Convenience sampling was finally employed to select participants who are easily accessible. The sampling technique allows the researchers to cherry-pick participants who can offer rich, in-depth insights into the GCDP implementation and its effect on residents’ well-being. The selection of these participants, who provide diverse perspectives and valuable information on the implementation level and effects of GCDP, was based on the six criteria outlined in the recruitment procedure section. The researchers chose to interview Lagos megacity residents living in areas with high or low GCDP implementation; Residents (Community leaders) actively promote sustainable practices; Lagos megacity Local government officials involved in green city projects; and Experts in BEPs and HMPs.

### Data collection

The investigator informed the participants of the study’s purpose, and they provided verbal consent. The author’s choice of BEPs, HMPs, and Residents as participants in the study is based on the GCDPs and their impact on the residents. The investigators conducted the interviews in English. As shown in Table 1, all fifteen (15) participants who participated in the exercise are residents of Lagos megacity, Nigeria. However, three (3) research assistants (M.Sc. Students) in the architecture department, Covenant University, Nigeria and the authors conducted the In-Depth Interviews (IDIs) in a controlled, systematic and organised manner on weekends (Friday through Sunday), in the mornings and evenings (9:30–12:30 am and 5:30–8:30 pm) over a six (6) month period amounting to 432hours. The study employed a narrative approach to reconcile conflicting accounts and highlight obstacles that could present opportunities for innovation, particularly in GCDP initiatives.

### Ethical considerations

Before data collection commenced, the application was submitted to the institutional review committee, the Covenant University Health and Research Ethics Committee (CHREC). CHREC reviewed the protocol and issued a formal waiver of full ethical approval, determining that the study presented no more than minimal risk and did not involve vulnerable populations. Written informed consent was obtained from all participants. The data collection process involved direct participation, so the authors conducted the in-depth interviews with experts. However, written informed consent forms were distributed to and obtained from participants. All procedures performed in this study were conducted in accordance with the 1964 Declaration of Helsinki and its later amendments.

### Data analysis

The authors developed observational notes and transcribed and analysed the data using thematic analysis. Coding followed the standard process of assigning short descriptive labels (codes) to segments of the qualitative data (the transcribed interviews) to identify recurring patterns and meanings. This systematic process began with step-by-step coding, familiarising oneself with the data, then generating initial codes by labelling key phrases or ideas, followed by searching for themes by grouping related codes, and finally refining these themes for clarity and significance to your research questions. However, to perform an inter-rater reliability (IRR) check, two independent coders manually coded the transcripts. Inter-rater reliability was assessed by comparing codes; disagreements were resolved through discussion and reconciliation. The authors deductively manually coded transcripts under pre-existing themes which were uncovered from literature as the obstacles to GCDP implementation (*Increase in Inhabitable Sites, Lack of Adequate Infrastructure, Zero to No Awareness of the Environment, Non-Awareness of GCDP Implementation Benefits, Lack of Protection for Natural Heritage Sites, Excessive generation of solid waste and poor wastewater management and Insufficient Policy Implementation Efforts*) which is in line with three objectives – Identifying the obstacles to GCDP implementation in Lagos, Nigeria, ascertaining the GCDP implementation level in Lagos, Nigeria, and assessing the effect of GCDP implementation on residents’ well-being in cities. The authors presented participants’ constructions of their experiences with the implementation of GCDP in Lagos through direct quotes and an ethnographic synopsis. The institutional ethics review committee (Covenant University) approved the ethical principles for qualitative research during and after data collection.

## Results and discussion

This section presents findings on the obstacles to GCDP implementation in Lagos, Nigeria, the implementation level of GCDPs in Lagos, Nigeria, and the effects of GCDPs on Lagos residents’ well-being.

### The obstacles to GCDP implementation in Lagos, Nigeria

In Lagos, achieving GCDPs has been only marginally successful. It is due to several obstacles: an increase in inhabitable sites, lack of adequate infrastructures, zero to no awareness about the environment, lack of protection for cultural/natural heritage, Non-Awareness of GCDP implementation Benefits, Excessive generation of solid waste, poor wastewater management and Insufficient Policy Implementation Efforts [60]. The investigators evaluated Lagos megacity based on the following criteria, which are in line with GCDPs – renewable energy, waste management strategies, water, landscape, gardens and biodiversity, sustainable transport, adoption of sustainable materials, green buildings and districts and use of passive design principles, housing types and conditions, intelligent systems and smart cities, urban governance, leadership and best practices, climate and context and green resilient infrastructure. The investigators used Lagos as a case study due to its status as a megacity and commercial capital. The Lagos Government’s agenda aims to improve the megacity’s environmental quality and to evaluate its effectiveness against GCDP criteria. However, the investigators discussed the identified obstacles to GCDP implementation as follows:

#### Increase in inhabitable sites.

**Fig 2:**
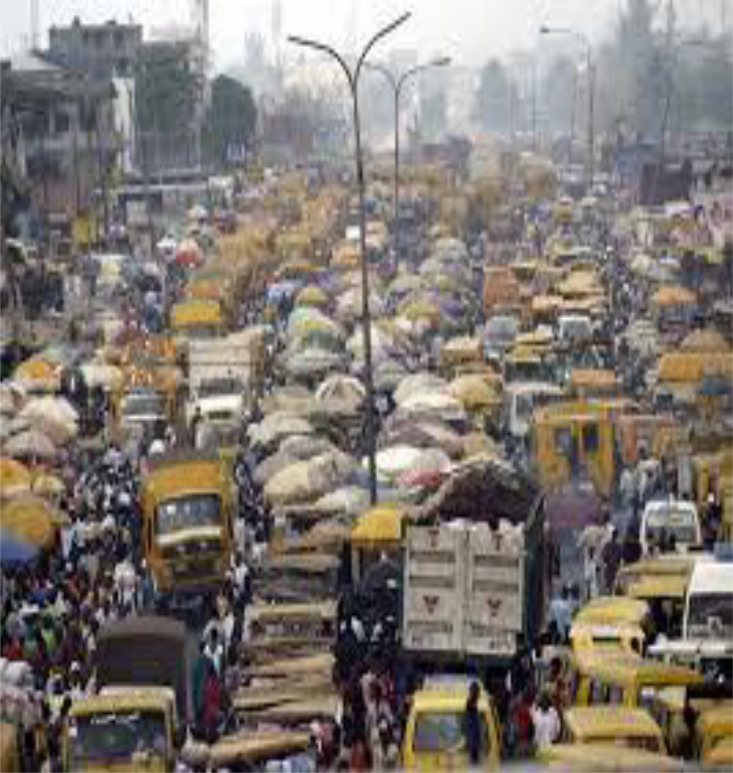
Random increase in population resulting in inhabitable space and overcrowding. Source: https://africanarguments.org/2012/02/diary-whatever-happened-to-africas-rapid-urbanisation-by-magnus-taylor/.

According to a recent report, Lagos, Nigeria, is the second-worst city to live in among 172 cities [61], as shown in Fig 2. The Economist Intelligence Unit (EIU), in its 2022 ranking of the most unliveable cities, ranked Lagos 171st out of 172 countries. Consistent with the In-Depth Interview (IDI) conducted:

*“Lagos, like most cities, has developed in an unchecked and unorganised way, which has resulted in the index of several glitches such as over-crowding, traffic congestion, environmental pollution, unemployment, over-population and deterioration of urban facilities, which have sequentially caused several other challenges confronting GCDP implementation in Nigeria like Ecological degradation, fraud, poverty, youth restlessness, political instability and Boko Haram insurgencies”.* ***(Clinical Psychologist, IDI: 2023)***

### Lack of Adequate infrastructure

**Fig 3:**
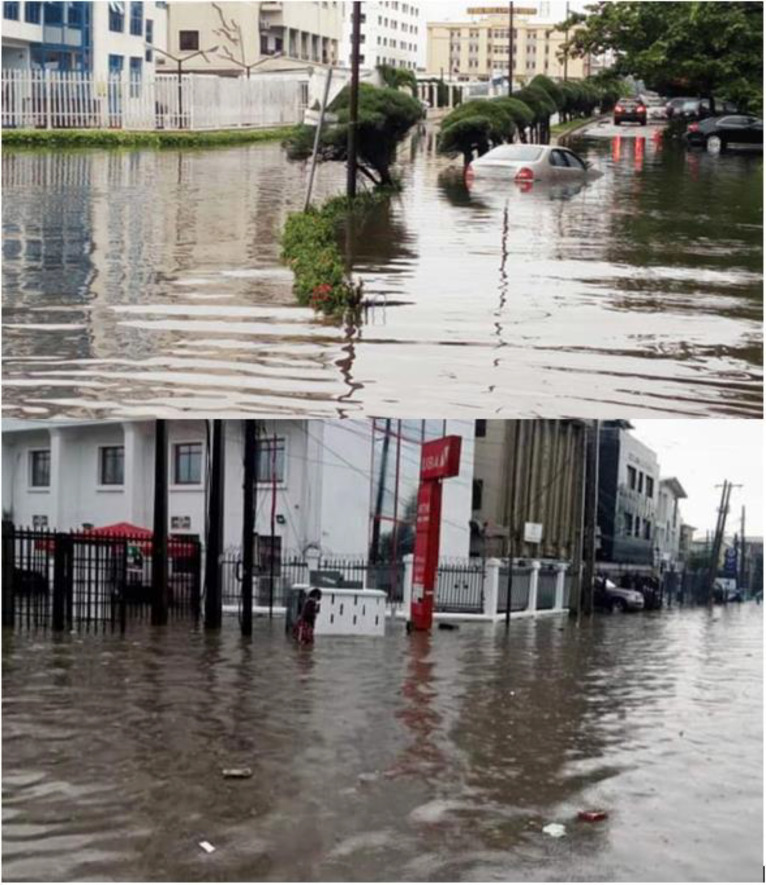
3a and b: A typical Lagos street with floods, depicting inadequate infrastructure. Source: https://www.ecohubmap.com/hot-spot/lagos-in-nigeria-risks-of-going-under-water/b0d2u2mlcf3tedv and https://humanglemedia.com/lagos-floods-victims-face-homelessness-loss-of-property-death/.

Lagos has many slums/squatter settlements constructed on the islands and mainland [62]. The city is tackling an eroding shoreline, making it susceptible to flooding, traceable to global warming and human-made action over a protracted period, as shown in Fig 3a and 3b. Lagos lacks functioning infrastructure provisions. Infrastructure facilities are inadequate to meet demand, causing overcrowding or amenity restrictions. All these hinder GCDP implementation. For this reason,

“*Lagos can only be greenly developed if the government concentrate on GCDP implementation, hindered by poor funding, environmental management and environmental management exclusion in the national development plan”.* **(Public health analysis, IDI: 2023)**

#### Zero to no awareness of the environment.

**Fig 4:**
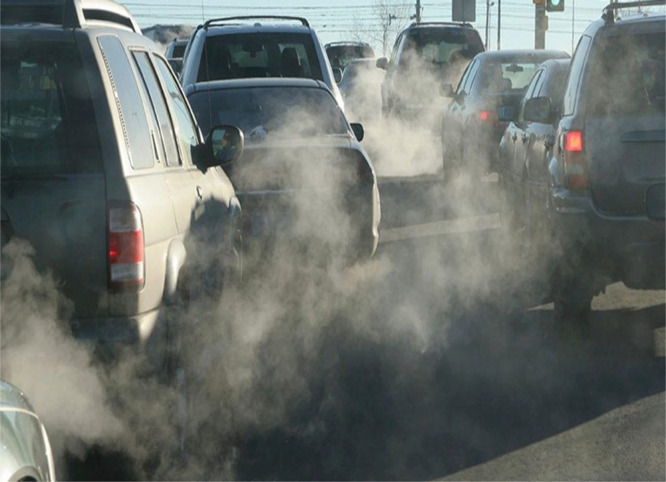
The impact of zero awareness in the Lagos environment. Source: https://www.environewsnigeria.com/vehicular-emissions-responsible-for-70-of-lagos-choking-smog-lasepa/.

Air pollution in Lagos is caused by poorly maintained and uncontrolled vehicles on the roads, as shown in Fig 4. Water pollution resulting from unplanned raw sewage disposal, sediment-laden overflow, and waste into the Lagoon creates health/well-being concerns [63,64]. Rapid urbanisation, housing shortages, poverty, and traffic in a growing city like Lagos require better transport solutions. The IDI with respondents revealed that:

*“The obstacles facing the concept of GCDP include providing a green water and energy supply, decreasing climate change and impacts adaptation, designing a pollution/waste-free future, generating effective, healthy, resilient cities, and promoting informed decisions/actions. The 3-R technique (reduce, reuse and recycle), which emphasises resource conservation, reusing rather than discarding products and commodity recycling, contributes significantly to achieving GCDP”.* ***(Well-being Architect, IDI: 2023)***

GCDP awareness means being informed about the natural surroundings and understanding how our actions affect the well-being of the local and global environments (nature, living and non-living). Residents’ awareness may have ranged from very low to very high, depending on socio-economic factors such as income, family members, education, age, and gender. **(Environmental Biologist, IDI: 2023)**

*To raise awareness of GCDP, we can avoid buying disposable items such as paper towels, plastic bottles, and plastic bags. Start a composting and recycling program in communities where there are none. It will help reduce waste production. It is essential to demonstrate how humans can protect and preserve their natural resources. Educating the population can reduce plastic and water waste while promoting recycling to reduce landfill waste.* **(Residents, IDI: 2023)**

*A lack of awareness is the failure to see beyond thoughts. It means we cannot grasp the impact of our emotions, actions, and behaviours. It can lead to waste accumulation in streets and public spaces, causing major environmental problems. Additionally, having a limited understanding of the causal relationship between human activities and environmental issues may hinder residents’ ability to address these problems effectively.* **(Residents, IDI: 2023)**

#### Lack of protection for natural heritage sites.

**Fig 5:**
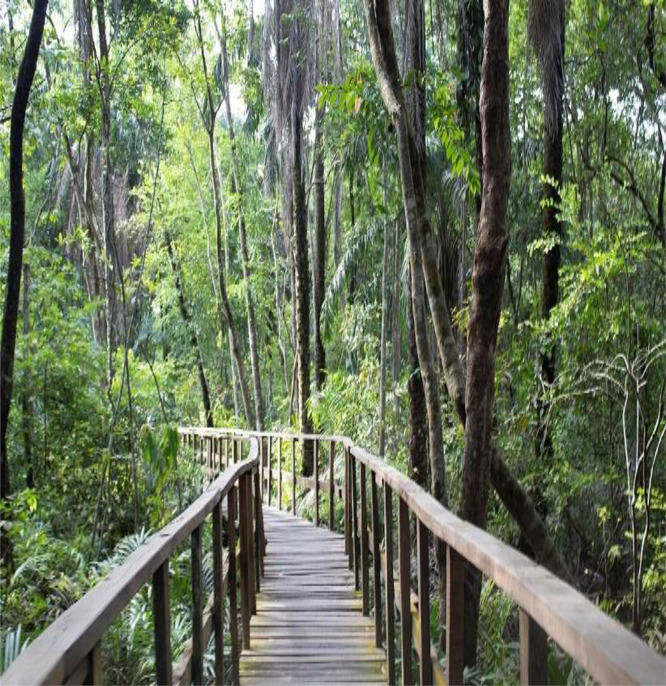
Lekki Conservation Centre, Lagos, showing cultural/natural heritage site. Source: https://en.wikivoyage.org/wiki/Lekki_Conservation_Centre.

The issues affecting unprotected natural heritage sites include violence, conflict, pollution, trafficking, unchecked urbanisation, and uncontrolled tourist development, as shown in Fig 5. Unprotected natural heritage sites may cause obstacles to GCDP implementation in Lagos. An architect said during an interview:

*“The barriers affecting GCDP implementation are knowledge, process, culture and management barriers. Today, Lagos, Nigeria, has experienced urban decay due to the absence or collapse of basic amenities”.* ***(Salutogentic Architect, IDI: 2023)***

#### Non-awareness of GCDP implementation benefits.

**Fig 6:**
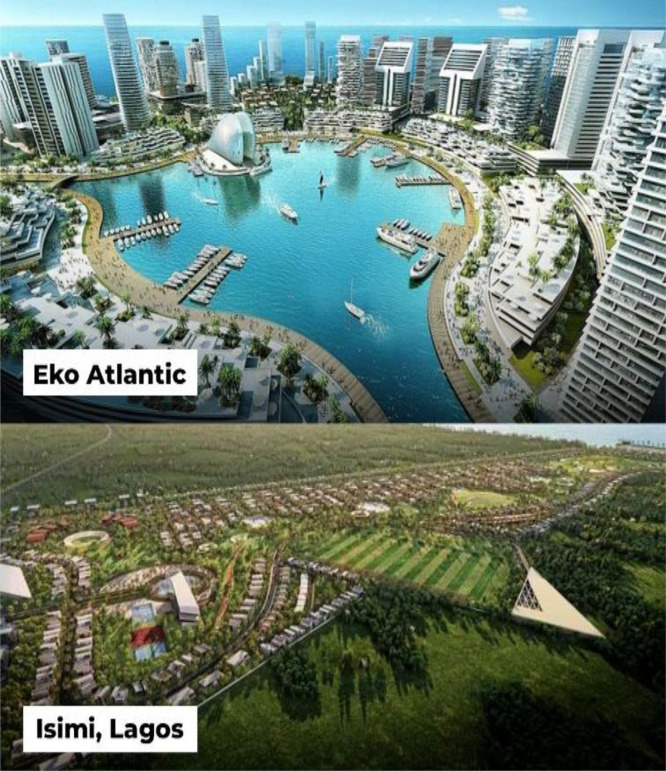
Lagos megacity adoption of GCDP implementation. Source: https://nairametrics.com/2023/05/17/eko-atlantic-city-and-isimi-lagos-two-modern-legacies-designed-by-the-same-architect/.

GCDP implementation in Lagos can improve the environment, guarantee rich biodiversity, lessen air pollution, ensure water storage, reduce noise, help cool in warm periods, and reduce psychosocial/physical health stress caused by noise, traffic jams, social density, frantic life rhythms, and densely packed public transport; see Fig 6. GCDP implementation is vital for a climate-proof, green environment, mitigating pollution and reducing the urban heat island effect [65–67]. Investment in green infrastructure can generate and sustain services in industries such as forestry, land administration and conservation. According to the investigator’s IDI

*“The obstacles to GCDP implementation are no awareness of GCDP benefits, ecological degradation, deficient policy implementation efforts, air and water quality, extreme solid waste generation, poor wastewater management, sanitation, suburban sprawl, climate change, energy use and cities’ ecological footprint”.* ***(Environmentalist, IDI: 2023)***

#### Excessive generation of solid waste and poor wastewater management.

**Fig 7:**
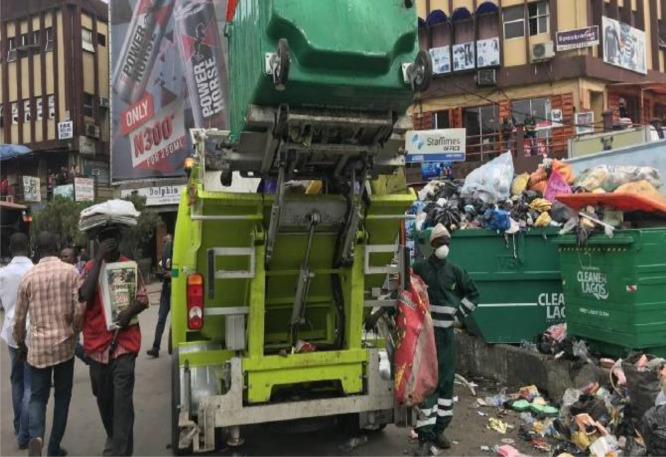
Lagos waste generation and management. Source: https://nairametrics.com/2018/04/18/lagos-state-government-sued-over-new-waste-management-law/.

Lagos megacity, home to over 24 million people, has identified over 13,000 metric tonnes of solid waste generated daily [68]. Fig 7 shows that continuous indiscriminate solid waste disposal is increasing and associated with poverty, bad governance, urbanisation, population growth, low standards of living, low green awareness, and inadequate management knowledge. IDI indicates that:

*“GCDP rely on three (3) interacting pillars: urban planning and transportation, energy and materials, and water and biodiversity. However, the regulations that can reduce prospects for a green city setup include zoning density standards, efficient waste management, storm sewer connection measures, and minimum parking and road widths.* ***(Therapeutic Architect, IDI: 2023)***

#### Insufficient policy implementation efforts.

**Fig 8:**
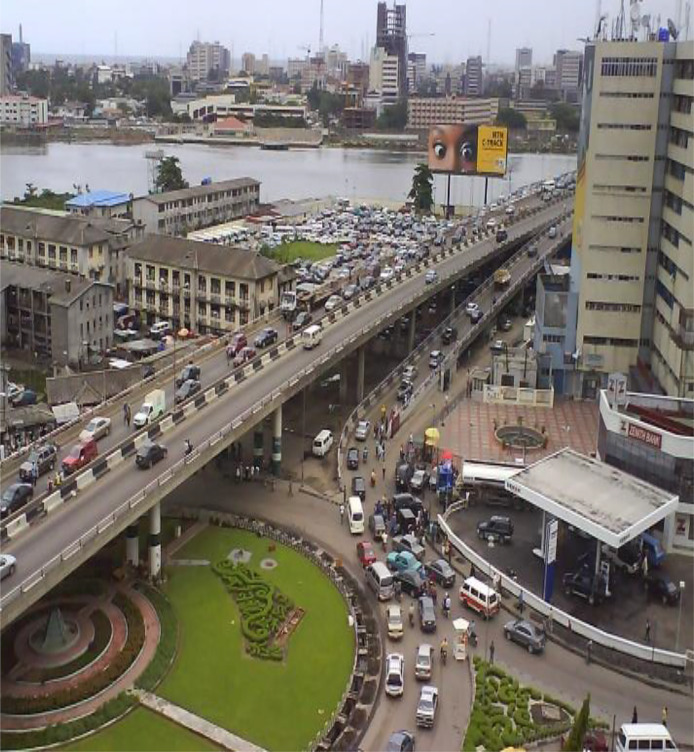
Policy implementation a key challenge to GCDP implementation. Source: https://businessday.ng/analysis/article/the-beautification-of-lagos-matter-arising/.

Fig 8 shows a panoramic view of a section of Lagos Megacity. However, some policy implementation challenges recognised in Lagos, Nigeria, include corruption, no government policies continuity, insufficient material resources, inadequate human resources, equipment and tools, legislation and lack of proper funding, incompetence and bad governance, leading to an implementation gap (distance between specified policy objectives and the accomplishment of such strategic objectives) [69,70]. The IDI report indicated that:

*“Among the main barriers identified to GCDPs implementation, funding was a lack of collaboration, no long-term thinking, insufficient expertise and knowledge related to financing solutions and no policy mandate to facilitate the uptake of finance”.* ***(Clinical Psychologist, IDI: 2023)***

### The GCDP implementation level in Lagos, Nigeria

Research showed that seventeen (17) GCDPs were identified from the literature, which experts and professionals interviewed in Lagos, Nigeria, corroborated. Since the study was qualitative research, all data and findings acquired, analysed, and presented were exploratory-descriptive, using words and images, following the research method. However, the investigators identified, evaluated and scored twelve (12) contest-specific GCDPs in the Lagos megacity for this purpose, as shown in Table 2. The investigators used a grading method to evaluate the level of greenness implementation in the Lagos megacity. It helped give greenness an implementation value in Lagos. The investigators graded the implementation level as follows: none = 0; high = 4. The researcher further converted the summation of the scores to percentages.

**Table 2:** Checklist to determine the implementation level of the twelve (12) contest-specific GCDPs in the Lagos Megacity.

SN	Green City Development Principles (GCDPs)	Description Of Green City Development Principles (GCDPs) Implementation Level In Lagos, Megacity	Greenness Value For Lagos Megacity				
			None (0), Low (1), Average (2), Slightly High (3), High (4)				
			0	1	2	3	4
1	Renewable Energy & zero CO2 emissions	• 84.2. Per cent of households in Lagos have access to electricity • Electricity supply is irregular, resulting in a low per capita electricity consumption of 0.8 gigajoules. • Regularised electricity supply in Lagos, Nigeria, will increase electricity consumption per capita and CO2 emissions from electricity consumption per person (kg/person), now at 983.9. • Therefore, clean energy sources will be like Oslo’s use – 65% renewable energy. • Renewable energy use in residential units in Lagos is [71,72].		✓			
2	Waste Management and Reduction Strategies	• Lagos megacity needs viable solid waste management. • Lagos generates about 12,000 metric tons of waste daily (0.72 kg/person/day). • Lagos State Waste Management Authority (LAWMA) is an agency with supervisory roles and statutorily charged with implementing, advocating, monitoring, and enforcing waste management policies. • The Lagos Megacity Project is the leading waste policy. • The waste-to-wealth programme initiatives have helped Lagos establish one of Africa’s largest compost plants, which converts 800 tonnes of solid waste into fertiliser daily. • Also, four small-scale plastic recycling plants convert 30 metric tonnes of recycled nylon or other plastics into usable products such as shopping bags [73,74]. • The Lagos State Government’s partnership with West Africa ENRG is gearing up to establish Nigeria’s first waste-to-energy facility, which will generate 25 megawatts of electricity [75]. Schemes include door-to-door household solid waste collection by the PSP, which covers about 63% of the Lagos megacity. In comparison, about 37% are seldomly serviced (such as Ikorodu, Epe, Badagry, Eti-Osa, Olorunda, Oto-Awori, etc.) [76].			✓		
3	Water Efficiency, Recycling and Stormwater Management	• Lagos has a water management emergency. • Only 10% of Lagos’s population has access to public water utilities. • Ninety per cent (90%) of Lagosians get water either from individual boreholes or informal sector participants (water vendors) [77]. • According to experts, the proliferation of private boreholes results in a landslide. Lagos has a water system leakage rate of 30.5%, compared with Latin American Index cities, which have the highest leakage rate worldwide at 35% [78]. • This water leakage causes contamination—Escherichia coli, Salmonella, Streptococcus, and Bacillus are contaminants in piped water—a few per cent of treated wastewater in Lagos [79].		✓			
4	Landscape, Gardens and Biodiversity	• The Lagos State Parks and Gardens Agency (LASPARK) demonstrated its commitment to enhancing the Lagos Greens through green renewal and programmes to achieve visually pleasing environs [80]. • The greenery programmes include creating, beautifying, and maintaining parks and gardens, as well as landscaping and open spaces. • Tree planting, maintenance, and the establishment of care and intervention systems are strategies to mitigate the effects of climate change and maintain the city’s biodiversity. • LASPARK has planted over 7 million trees across Lagos and established 11 Public Events Parks and 297 Gardens [81]. • According to [82], a 2017 survey of 1560 residents across the city indicated that public parks are green spaces, but are few, ill-equipped, and in poor condition.			✓		
5	Sustainable Transport	• With over six million cars on Lagos roads daily, routes are congested and polluted. • The Lagos public transport system consists of thousands of secretly acquired buses [14]. • Water transportation in Lagos is a greener alternative to congested road transport. According to [83], a ferry has lower carbon emissions per passenger than a coach or vehicle. • In Lagos, water transport has yet to make an impact due to reliance on road transport.		✓			
6	Adoption of Sustainable Materials	• [84] claimed that poor data and expertise in green construction practices are the primary limits to adopting sustainable construction in Lagos.		✓			
7	Green Buildings & Districts & Passive Design Principles	• Lagos has accepted the International Finance Corporation (IFC) green building initiative to ensure green buildings in the megacity [85].		✓			
8	Housing Types and Conditions	• The inadequacy of decent residential accommodation has resulted in 42 slum areas in the Lagos megacity [86,87].		✓			
9	Intelligent Systems and Smart Cities	• [88] assessed the current position of Lagos concerning smart city indicators as not measuring up to the international best practices model of an intelligent city. • However, there is the Lagos Metro and Smart City Initiative, with the ongoing laying of 3,000 km of fibre metro network cables and a broadband framework to enable speed in commercial activities [89].		✓			
10	Urban governance, leadership, and best practices	• In Lagos, there are still cases of environmental abuse. However, the Lagos megacity has made compliance with ecological law sacrosanct by establishing several enforcement agencies [90–92].		✓			
11	Climate and Context	• The greenery programmes include operational parks and gardens, landscaping and beautification of open spaces, tree planting and maintenance, and the establishment of support and intervention systems to mitigate the effects of climate change and maintain biodiversity in the Lagos megacity.			✓		
12	Green, Resilient Infrastructure	• Lagos streets flood due to the inefficient daily dumping of 6,000–10,000 tonnes of garbage. [93]. • After a rainstorm, garbage piles up in open gutters and covers streets [94].		✓			

From the result in Table 2, all twelve (12) GCDP parameters show that the implementation level in the Lagos megacity is in the range of low (1) and average (2) due to several factors. Nine (9) of the 12 GCDP parameters are in the low (1) category, and the remaining three (3) [Waste Management and Reduction Strategies, Landscape, Gardens and Biodiversity, and Climate and Context] are in the average (2) category. As explained in Table 2 under the description of the GCDP implementation level column, the government and residents have attempted to implement these principles.

However, in the implementation level 3-slightly high and level 4-high, none of the GCDPs parameters can measure up to the values because, according to the global grading standard, only twelve (12) parameters identified in Lagos Megacity did not measure up to the levels 3 and 4 categories. Hence, the non-inclusion in the table. The same applies to the none (0) value category. To include a non-zero (0) value is to add the remaining five (5) GCDP parameters (1. Density and retrofitting of existing districts, 2. Using passive design principles, 3. Cultural heritage, identity and sense of place, 4. Local food and short supply chains, and 5. Education, research, knowledge, and strategies for cities in developing countries are not mentioned in the table. Adding these five parameters would mean there would be no description column for them.

### The effect of GCDP implementation on Lagos residents’ well-being

Contact with nature is vital and beneficial to humans through GCDP implementation, as evidenced by the scientific literature. GCDP is associated with reduced mortality and better psychosocial well-being. Social contact, physical activity, maintenance, and access to urban green infrastructure foster a relationship between GCDP and residents’ health/well-being. GCDP implementation may help deprived populations, reduce health disparities, and mitigate the adverse effects of built-environment concerns. The impact of environmental degradation on human health can range from death to psychosocial problems, including those resulting from air pollution and noise [95]. GCDP can restore ecologically damaged urban areas, ensure decent, affordable housing for all social groups, improve job opportunities for disadvantaged groups, such as women and minorities, and support local agriculture and future progress and expansion. GCDP benefits the planet by saving money, time, and resources, enabling generations to access clean air and water.

**Fig 9:**
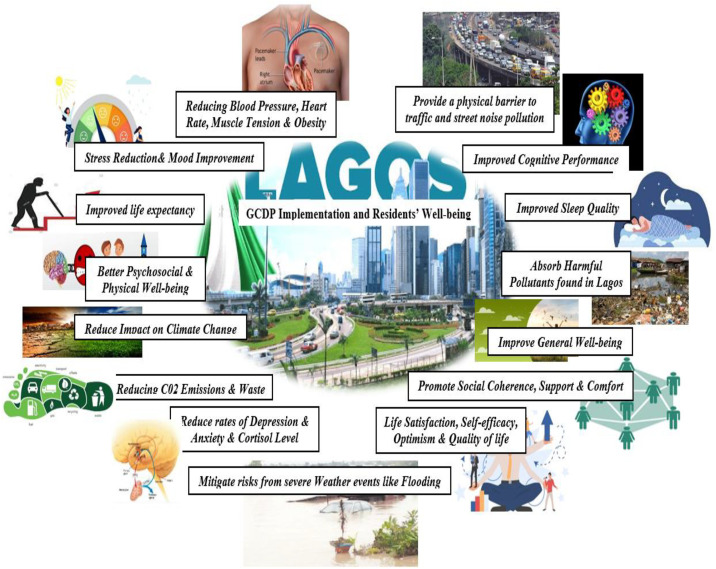
The GCDPs implementation level and effect on residents’ well-being.

To this end, the GCDP implementation level in Lagos has impacted megacity residents’ well-being in the following ways, as shown in Fig 9: Studies have shown that GCDP can ***reduce rates of depression and anxiety, reduce cortisol levels and improve the general well-being*** of Lagos residents**.** GCDPs consider the setting, create vibrant community spaces, and support sustainable development while reducing CO^2^ emissions and waste to enhance residents’ well-being. GCDPs improve the environment, support biodiversity, reduce air pollution, enhance water storage, dampen noise, and aid cooling during warm periods. They are vital to a climate-proof, green environment. GCDP can promote ***better psychosocial and physical well-being,*** thereby reducing morbidity and mortality among Lagos megacity residents by creating psychosocial relaxation, Stress reduction, and mood improvement, assisting physical activity, and decreasing exposure to air pollution. GCDPs reduce carbon and other pollution in the atmosphere, increase residents’ well-being by providing community spaces and ***mitigate risks from severe weather events like flooding***. The in-depth interviews conducted in Lagos megacity, Nigeria, demonstrated the well-being benefits of GCDPs in the living environment, emphasising that parks can help fight diabetes and ***improve cognitive performance***. Lagos communities intend to implement GCDP to enhance residents’ physical exercise, social interactions, relaxation, and mental restoration.

Exposure to GCDP implementation benefits residents’ well-being by reducing chronic diseases, improving psychosocial health and pregnancy outcomes, and improving ***life expectancy*.** The prioritisation of GCDPs results in cleaner air for breathing. GCDP implementation in Lagos has encouraged outdoor activity, ***Improved sleep quality*** and prevented associated chronic diseases. The Level of GCDP implementation in Lagos has affected residents’ sense of social support and stimulation. Air and water pollution in Lagos pose health and safety risks that affect residents’ well-being. GCDP implementation has ***absorbed harmful pollutants found in Lagos*** and ***reduced the impact of climate change*** to a manageable level. GCDP implementation in Lagos can enhance residents’ emotions and well-being by ***lowering blood pressure, heart rate, muscle tension and obesity***. GCDP implementation in Lagos, Nigeria, has facilitated interactions and ***promoted social cohesion, support and Comfort*** among residents. The implementation of the GCDP in Lagos can ***provide a physical barrier to traffic and street noise pollution***. Currently, factors affecting the megacity residents’ well-being are stress, pessimism, negative mood, depression, anxiety, loneliness, sleeplessness, and fatigue. However, at the GCDP implementation level, some positive factors include life satisfaction, self-efficacy, optimism, **and quality of life** [96]. However, Table 3 presents a summary of the findings from the Human participants in the study.

**Table 3:** Summarising the key findings derived by human participants.

SN	Human Participants	Key Findings
1	HMPs interviews	reveals a strong link between green infrastructure and residents’ self-perceived health benefits, emphasising the need for improved access and quality of these spaces.
2	BEPs	underscore the need for a comprehensive approach that integrates GCDP principles into urban planning and development to ensure a more livable and environmentally sustainable city for all residents
3	Residents	Green infrastructure is perceived as beneficial for well-being and community, but its poor quality and limited access negatively impact these perceptions. Precisely, residents value green spaces such as parks and gardens, but access to and quality of these spaces vary significantly across neighbourhoods.
4	Lagos megacity Local government officials	emphasised the relevance of green infrastructure for a sustainable and healthy Lagos, but also highlighted the urgent need to address the current limitations in its provision and management.

## Discussion

The green development propositions in the Braundlandt Commission report and the Rio de Janeiro Earth Summit Agenda 21 – focus on solving environmental problems emanating from development [97]. GCDP is a goal of urban development [98]. Environmental issues within Lagos’ megacities pose serious health threats to urban residents. For the above reasons, the study aims to investigate the effect of the level of implementation of GCDP as a development model on enhancing residents’ well-being in the Lagos megacity, Nigeria. In Lagos, achieving GCDPs has been slow and steady owing to several obstacles [99]. However, the data collected and analysed show that of the seventeen GCDPs identified, twelve (12) contest-specific GCDPs apply to the Lagos megacity. The investigators evaluated the twelve GCDPs to assess the level of implementation using the greenness-values grading scale. The study identifies and analyses the barriers to, and the implementation level of, GCDP in the Lagos megacity, and the relationship between the implementation level of GCDP and the well-being of Lagos megacity residents. Therefore, the findings specified the effects of the GCDP implementation on Lagos residents’ well-being, as highlighted in Fig 9.

In a similar study, [100] explained that an innovative aspect of GCDP implementation in Indonesia is the elaboration of green maps by local communities. Green maps provide complete information about green sites and infrastructure to promote a green urban lifestyle and inspire the urban greening movement. In this regard, GCDP is an indicator of planning reform in Indonesia, dedicated to creating linkages among policy, planning, design and implementation. It is an adaptive planning intervention, striving to control the negative externalities propagated by potentially destructive urbanisation forces. As a responsive tool, GCDP ensures pro-green place-making [101].

Furthermore, [102] asserted that in the Global North, green city development concepts are incremental to mainstream green infrastructure and nature-based solutions in supporting ecosystem services. Conversely, in Global South cities, due to rapid and extensive urbanisation, the green city is adopted only in a fragmented manner. Previous studies have outlined three factors (urbanisation, biophysics, and governance) that underlie differences in GCD approaches between the Global North and the Global South. Still, more studies are needed to explain how these factors will shape the green-city trajectory in Global South cities. Hence, [103] believe there is a misunderstanding of the GCD approach by actors at the national or local level. There is no local commitment and capacity to undertake the program in cities. [104] believe that rapid urbanisation in the Global South is the cause of severe social, economic and environmental challenges for future urban development. They stated that Gaborone, Botswana’s capital, is a primate city that faces the same challenges as megacities. Since GCDP is the only development alternative for Gaborone, there is a need to improve collaboration through existing planning instruments at the national and local levels. Also, [105] agrees that, as part of the recommendation for the Global South to keep up with GCDP, stakeholders’ effective participation supports policies that encourage citizens to take full ownership of urban development. It facilitates effective implementation with the potential to improve the quality of life in Lebanon. However, [106] clarified that GCD leads to the creation of a livable city ecosystem. The implementation of GCDP in Malang is on the Malang City Green Open Space (GOS) Master Plan 2012–2032. One of Malang GCDP’s strategic efforts is to realise a livable urban GOS by improving the quality of urban resilience.

In a related study, [107] describes and analyses community participation in the Green City Action Plan (GCAP) through the Green Community Forum in Tulungagung. GCDP is an adaptation strategy that responds to the impacts of climate change. Three elements determine the growth and development of community participation in the Global South: opportunity, will, and ability. [108] analysed and evaluated city changes in Poland based on selected GCD characteristics. The improvement in ecologisation was achievable by implementing GCDP solutions in the urban areas of Poland. In assessing the effect of GCDP implementation on residents’ well-being in cities, [109] appraise GCD characteristics in the Sheikh Tappeh neighbourhood in Urmia. The World Health Organisation introduced the concept of green city indicators (GCI). Based on the collected data, the health indicators of the Sheikh Tappeh neighbourhood showed a significant relationship between cultural knowledge and favourable cultural conditions. Still, other health indicators are far from optimal conditions.

## Conclusion

The study investigated the implementation levels of GCDPs accessible in Lagos, Nigeria, to ascertain the current implementation level within the study area. The study investigated principles found in previous literature. The implementation levels are relatively low in Lagos (about 36%). Based on the findings, a high level of implementation of the GCDPs in the study area and among relevant stakeholders in the building industry should provide platforms for promoting the benefits of green development in Nigeria. Furthermore, global and regional collaboration is needed to complement national and local actions. Thus, at the local level, the Lagos megacity has made compliance with an Environmental Impact Assessment (EIA) report a vital condition for approving all developments. The enforcement of periodic “environmental auditing” of existing industrial establishments. The establishment of the Department of Environment, with relevant human resources within the Town Planning Authorities. The enforcement of laws against illegal structures and illegal conversion of open spaces, recreational parks and greenbelts in Lagos into unauthorised development. Like the Green City Volunteer Network, the Lagos megacity has established a committee to educate people about the importance of GCDPs. And the enforcement of compliance with waste management regulations in the Lagos megacity by the relevant agency.

## Limitations of the study

The study offers robust perceptions of the implementation level of GCDP and its effects on megacity residents’ well-being. It explores varied perspectives on the effects of the level of GCDP implementation on megacity residents’ well-being. However, the research has some limitations. First, the results from IDIs and observation schedules might not capture all the effects of the level of GCDP implementation on megacity residents’ well-being in the Global South. Purposive sampling could present selection bias and limit the generalisability of the results. Second, lived experiences, given residents’ years of residence and professionals’ expertise, might affect the research outcomes. It may lead to underreporting of some obstacles to GCDP implementation through best green policy initiatives and practices, or to a tilted depiction of their experiences. Third, participants may have difficulty remembering certain details of their lived experiences due to the timing of the interviews. It can affect the data’s reliability and validity. Fourth, the list of participants shows a very high level of education. Since environmental issues tend to affect the lower socio-economic class, these participants’ views were not elicited. Omitting this group of people tends to introduce bias into the findings. Lastly, the study offers a rich understanding but may be deficient in statistical meaning. It can be attractive to quantify the effects of GCDP implementation on megacity residents’ well-being in Lagos, Nigeria, using the observation schedule from the case studies and interview methods. Therefore, mixed-methods research can provide a more inclusive understanding of the subject under study.

## Contributions of the study

The study highlights how environmental problems within cities/megacities in the Global South affect urban residents’ well-being. Continuous industrial emissions and other adverse environmental impacts may impede GCDP implementation in cities (e.g., Lagos Megacity). It indicates the need for adequate attention to environmental problems arising from development (across international, national, and local borders). Therefore, the GCDP implementation section of the law requires global and regional collaboration to complement state and local actions. It emphasised that the issue of illegal structures and illegal conversion of open spaces, recreational parks and greenbelts into unauthorised development needs to be taken seriously. Non-awareness of GCDP implementation benefits should not be an excuse for non-compliance by any developers of illegal structures. It used the activities of countries with the best green city policy initiatives and practices in GCDP implementation to suggest that all stakeholders should pay more attention to awareness and education on green city development principles in cities (especially Lagos Megacity).
