# Supplementary material for: Green city development principles (GCDP) implementation level and its effects on lagos megacity residents’ well-being, Nigeria: A qualitative study
Source: PLoS One. 2026 Aug 4;21(8):e0354733. doi: 10.1371/journal.pone.0354733 (PMC13436778; doi:10.1371/journal.pone.0354733)
Supplement: Supplementary file 3 [file pone.0354733.s004.docx]

**#Author Response to Reviewer**

**Manuscript ID**: PONE-D-25-25206R2

**Manuscript title**: Green City Development Principles (GCDP) Implementation Level and its Effects on Lagos Megacity Residents’ Well-being, Nigeria: A Qualitative Study

**Journal**: PLOS One

**Article type**: Original Research

**Keywords:** Green Development, City Development Principles, Urbanisation, Planning Models, Green Cities, Residents’ Well-being

**Corresponding Author:** Eghosa N. Ekhaese

**Decision:** Minor Revision (conditional acceptance)

**Editor Comments**

**Journal Requirements:**

If the reviewer comments include a recommendation to cite specific previously published works, please review and evaluate these publications to determine whether they are relevant and should be cited. There is no requirement to cite these works unless the editor has indicated otherwise.

**Authors’ Response**

Noted and thank you for the privileged information

**Editor Comments**

Please review your reference list to ensure that it is complete and correct. If you have cited papers that have been retracted, please include the rationale for doing so in the manuscript text, or remove these references and replace them with relevant current references. Any changes to the reference list should be mentioned in the rebuttal letter that accompanies your revised manuscript. If you need to cite a retracted article, indicate the article’s retracted status in the References list and also include a citation and full reference for the retraction notice.

**Authors’ Response**

Again, the authors are grateful for comments and thank you for the information.

**Additional Editor Comments: OVERALL ASSESSMENT**
The authors have responded substantively to the required revisions from the previous round. I acknowledge that they have:
• Clarified the literature selection method briefly.
• Added explicit ethical consideration details (CHREC review, written informed consent).
• Elaborated on the six-step thematic analysis process.
• Expanded the limitations section to address participant selection bias.
• Confirmed compliance with image permissions and data sharing.

**Authors’ Response**

The authors are very grateful for your comments and detailed observations on how to improve the work. Thank you so much for your remarks. However, we promise to provide quality responses to all the issues. Thank you once again.

**Editor Comments**

The manuscript is now substantially improved in methodological transparency and scholarly rigour. However, three discrete issues remain unresolved. These are minor and can be corrected without re-review.

**Authors’ Response**

Noted and thank you for the comment and suggestion.

**Editor Comments**

**UNRESOLVED ISSUES (MINOR, REQUIRING FINAL CORRECTIONS)**

***1. Ethical approval statement remains internally inconsistent***

In the revised manuscript (pages 66-67), the authors state: “the application was sent to CHREC… it was returned to us that we need no ethical certificate because it was social research.” Then they write: “ethical approval was not needed because there was no high risk, no vulnerable group involved.”
This is problematic. A formal waiver of ethical review is still an ethics determination. The correct phrasing should be: “CHREC reviewed the protocol and issued a formal waiver of full ethical approval, determining that the study presented no more than minimal risk and did not involve vulnerable populations. Written informed consent was obtained from all participants.”
**Required action:** Revise the Ethical Considerations subsection to explicitly state that a waiver was granted, not that approval was “not needed.” Provide the date of the CHREC waiver determination if available.

**Authors’ Response**

Noted and thank you for the comment and suggestion. The authors have made corrections in the revised manuscript.

**2.** ***Thematic analysis*** – no evidence of inter-rater reliability process in the manuscript
In the author’s response, they mention two coders and an IRR check. However, the revised manuscript (page 67) does not include this detail. It only describes the six-step process generically.
**Required action:** Add one sentence to the Data Analysis subsection stating: “Two independent coders manually coded the transcripts. Inter-rater reliability was assessed by comparing codes; disagreements were resolved through discussion and reconciliation.”

**Authors’ Response**

Again, thank you for the comments and suggestions. Although we mention *that “two independent coders were used to code the same set of data (transcripts) using the same coding scheme. After coding, the codes were compared to find areas of agreement and disagreement. Thereafter, discussions and reconciliation were carried out to conclude the process”*, in the revised manuscript sent before,

However, we have restructured the sentence to fit the required narrative in the revised manuscript. Thank you.

***3. Raw qualitative data not verifiably shared***

The authors state that supplementary files have been attached. However, in the reviewed submission files, no de-identified interview transcripts or codebook are present. PLOS ONE requires the minimal data set (or a clear explanation of restrictions).
**Required action:** Either:

• Upload de-identified transcripts (or a representative sample) as supporting information, or
• Deposit them in a public repository (e.g., Figshare, Zenodo) and provide the DOI, or
• Provide a written statement from CHREC prohibiting sharing, with contact information for data access requests.

Failure to do so will result in rejection under PLOS ONE’s data availability policy.

**Authors’ Response**

Again, thank you for the comments and suggestions. I am not sure what this last means. However, the authors have addressed this subject in the revised manuscript.

**Editor Comments**

**WHAT THE MANUSCRIPT NOW CORRECTLY CONTRIBUTES**

• A context-specific qualitative assessment of GCDP implementation in a major African megacity.
• Clear identification of structural, environmental, and policy-related obstacles.
• A graded checklist of 12 contest-specific GCDPs with implementation levels.
• Direct quotes and thematic findings linking green infrastructure to resident well-being.

**Authors’ Response**

Again, we appreciate your comments. The authors are sincerely grateful for the succinct summary of the contributions.

**Editor Comments**

What remains appropriately caveated:

Selection bias (a highly educated sample), recall bias, limited generalizability, and a lack of statistical inference – all are now acknowledged as limitations.

**Authors’ Response**

Again, the authors are thankful for your comments and observations. Thank you sincerely for the detailed statement.

**Editor Comments**

**RECOMMENDATION**
Minor Revision. The manuscript is not acceptable in its current form, only due to the three issues above. These can be corrected within 7 days without external review.
Once the authors:
1. Revise the ethics statement to specify a formal waiver from CHREC.
2. Add the inter-rater reliability sentence to the Data Analysis subsection, and
3. Either share de-identified raw data or provide a documented ethical restriction with access contact information,

I will recommend acceptance.
No further methodological, statistical, or interpretive revisions are required.

**Authors’ Response**

Again, the authors are thankful for your scholarly and robust comments

**FINAL REMARK TO THE AUTHORS**

You have transformed a descriptively rich qualitative study into one that meets PLOS ONE’s standards for transparency and rigour. The remaining issues are administrative and easily corrected. Address them promptly, and this paper will make a useful contribution to the literature on green urbanism in Global South megacities.

Decision: Minor Revision (7 days)

The authors are sincerely grateful for the additional comments from the editor that helped improve this work. Thank you so much.
