# Supplementary material for: Green city development principles (GCDP) implementation level and its effects on lagos megacity residents’ well-being, Nigeria: A qualitative study
Source: PLoS One. 2026 Aug 4;21(8):e0354733. doi: 10.1371/journal.pone.0354733 (PMC13436778; doi:10.1371/journal.pone.0354733)
Supplement: Supplementary file 4 [file pone.0354733.s002.docx]

**#Authors' response to Journal Requirements-PLOS ONE Decision**

**PLOS ONE Decision: Revision required [PONE-D-25-25206] [EMID:1894e8cb8cae62b5]**

**Journal Requirements:**

When submitting your revision, we need you to address these additional requirements.

**Authors’ Response**

The authors appreciate your comments, observations, suggestions, recommendations, and advice on improving the work. Thank you so much for your positive words. However, we promise to provide quality responses to all the issues. Thank you once again.

1.Please ensure that your manuscript meets PLOS ONE’s style requirements, including those for file naming. The PLOS ONE style templates can be found at

<https://journals.plos.org/plosone/s/file?id=wjVg/PLOSOne_formatting_sample_main_body.pdf> and

[https://journals.plos.org/plosone/s/file?id=ba62/PLOSOne_formatting_sample_title_authors_affiliations.pdf](https://track.editorialmanager.com/CL0/https:%2F%2Fjournals.plos.org%2Fplosone%2Fs%2Ffile%3Fid=ba62%2FPLOSOne_formatting_sample_title_authors_affiliations.pdf/1/010f019818677d42-04be412e-7a5d-4fbc-a51b-ec94a195e13a-000000/WqMZfBtkJSsvqT3MA8pZW4Bx7J5fYp5u-ebQuwamA1c=220)

**Authors’ Response**

Noted and thank you for the information. We have complied with the necessary instructions.

2. We noticed you have some minor occurrence of overlapping text with the following previous publication(s), which needs to be addressed: - DOI 10.3389/frsc.2025.1506672

In your revision ensure you cite all your sources (including your own works), and quote or rephrase any duplicated text outside the methods section. Further consideration is dependent on these concerns being addressed.

**Authors’ Response**

Noted and thank you for the information. The authors have complied with the necessary instructions.

3. Thank you for stating the following in your Competing Interests section: “12975”

Please complete your Competing Interests on the online submission form to state any Competing Interests. If you have no competing interests, please state “The authors have declared that no competing interests exist.”, as detailed online in our guide for authors at <http://journals.plos.org/plosone/s/submit-now>

 This information should be included in your cover letter; we will change the online submission form on your behalf.

**Authors’ Response**

Noted and thank you for the information. We have complied with the necessary instructions.

4. Please ensure that you refer to Figures 2-8 in your text as, if accepted, production will need this reference to link the reader to the figure.

**Authors’ Response**

Noted and thank you for the information. The authors have complied with the necessary instructions.

5. Please include captions for your Supporting Information files at the end of your manuscript, and update any in-text citations to match accordingly. Please see our Supporting Information guidelines for more information: [http://journals.plos.org/plosone/s/supporting-information](http://track.editorialmanager.com/CL0/http:%2F%2Fjournals.plos.org%2Fplosone%2Fs%2Fsupporting-information/1/010f019818677d42-04be412e-7a5d-4fbc-a51b-ec94a195e13a-000000/95I2yH1kcpv32CY9dEaaL5oeHPnoeN9tXO_RWQalgL0=220).

**Authors’ Response**

Noted and thank you for the information. We have complied with the necessary instructions.

6. We note that Figures 2-4 includes an image of a participant in the study.

As per the PLOS ONE policy (<http://journals.plos.org/plosone/s/submission-guidelines#loc-human-subjects-research>) on papers that include identifying, or potentially identifying, information, the individual(s) or parent(s)/guardian(s) must be informed of the terms of the PLOS open-access (CC-BY) license and provide specific permission for publication of these details under the terms of this license.

**Authors’ Response**

Noted and thank you for the information. We have complied with the necessary instructions.

Please download the Consent Form for Publication in a PLOS Journal (<http://journals.plos.org/plosone/s/file?id=8ce6/plos-consent-form-english.pdf>). The signed consent form should not be submitted with the manuscript but should be securely filed in the individual’s case notes. Please amend the methods section and ethics statement of the manuscript to explicitly state that the patient/participant has provided consent for publication: “The individual in this manuscript has given written informed consent (as outlined in PLOS consent form) to publish these case details”.

If you are unable to obtain consent from the subject of the photograph, you will need to remove the figure and any other textual identifying information or case descriptions for this individual.

**Authors’ Response**

Noted and thank you for the information. The authors have complied with the necessary instructions.

7. We note that Figures 2-8 in your submission contain copyrighted images. All PLOS content is published under the Creative Commons Attribution License (CC BY 4.0), which means that the manuscript, images, and Supporting Information files will be freely available online, and any third party is permitted to access, download, copy, distribute, and use these materials in any way, even commercially, with proper attribution. For more information, see our copyright guidelines: <http://journals.plos.org/plosone/s/licenses-and-copyright>.

**Authors’ Response**

Noted and thank you for the information. The authors have complied with the necessary instructions.

 We require you to either (1) present written permission from the copyright holder to publish these figures specifically under the CC BY 4.0 license, or (2) remove the figures from your submission:

 1. You may seek permission from the original copyright holder of Figures 2-8 to publish the content specifically under the CC BY 4.0 license.

 We recommend that you contact the original copyright holder with the Content Permission Form (<http://journals.plos.org/plosone/s/file?id=7c09/content-permission-form.pdf>) and the following text: “I request permission for the open-access journal PLOS ONE to publish XXX under the Creative Commons Attribution License (CCAL) CC BY 4.0 (<http://creativecommons.org/licenses/by/4.0/>).

**Authors’ Response**

Noted and thank you for the information. The authors have complied with the necessary instructions.

Please be aware that this license allows unrestricted use and distribution, even commercially, by third parties. Please reply and provide explicit written permission to publish XXX under a CC BY license and complete the attached form.”

Please upload the completed Content Permission Form or other proof of granted permissions as an “Other” file with your submission.

**Authors’ Response**

Noted and thank you for the information. The authors have complied with the necessary instructions.

In the figure caption of the copyrighted figure, please include the following text: “Reprinted from [ref] under a CC BY license, with permission from [name of publisher], original copyright [original copyright year].”

**Authors’ Response**

Noted and thank you for the information. The authors have complied with the necessary instructions.

The authors have complied with the necessary instructions.

 2. If you are unable to obtain permission from the original copyright holder to publish these figures under the CC BY 4.0 license or if the copyright holder’s requirements are incompatible with the CC BY 4.0 license, please either i) remove the figure or ii) supply a replacement figure that complies with the CC BY 4.0 license. Please check copyright information on all replacement figures and update the figure caption with source information. If applicable, please specify in the figure caption text when a figure is similar but not identical to the original image and is therefore for illustrative purposes only.

**Authors’ Response**

Noted and thank you for the information. The authors have complied with the necessary instructions.

8. We note that your Data Availability Statement is currently as follows: All relevant data are within the manuscript and in Supporting Information files.

Please confirm at this time whether or not your submission contains all the raw data required to replicate the results of your study. Authors must share the “minimal data set” for their submission. PLOS defines the minimal data set to consist of the data required to replicate all study findings reported in the article, as well as related metadata and methods (<https://journals.plos.org/plosone/s/data-availability#loc-minimal-data-set-definition>).

For example, authors should submit the following data:

- The values behind the means, standard deviations and other measures reported;

- The values used to build graphs;

- The points extracted from images for analysis.

**Authors’ Response**

Yes, the authors confirm that our submission contains all the raw data required to replicate the results of your study.

Authors do not need to submit their entire data set if only a portion of the data was used in the reported study. If your submission does not contain these data, please either upload them as Supporting Information files or deposit them to a stable, public repository and provide us with the relevant URLs, DOIs, or accession numbers. For a list of recommended repositories, please see <https://journals.plos.org/plosone/s/recommended-repositories>.

**Authors’ Response**

Noted and thank you for the information. The authors have complied with the necessary instructions.

If there are ethical or legal restrictions on sharing a de-identified data set, please explain them in detail (e.g., data contain potentially sensitive information, data are owned by a third-party organization, etc.) and who has imposed them (e.g., an ethics committee). Please also provide contact information for a data access committee, ethics committee, or other institutional body to which data requests may be sent. If data are owned by a third party, please indicate how others may request data access.

**Authors’ Response**

Noted and thank you for the information. The authors have complied with the necessary instructions.

9. If the reviewer’s comments include a recommendation to cite specific previously published works, please review and evaluate these publications to determine whether they are relevant and should be cited. There is no requirement to cite these works unless the editor has indicated otherwise.

**Authors’ Response**
Noted and thank you for the comment. The authors have complied with the necessary instructions.
