# Supplementary material for: Green city development principles (GCDP) implementation level and its effects on lagos megacity residents’ well-being, Nigeria: A qualitative study
Source: PLoS One. 2026 Aug 4;21(8):e0354733. doi: 10.1371/journal.pone.0354733 (PMC13436778; doi:10.1371/journal.pone.0354733)
Supplement: Supplementary file 5 [file pone.0354733.s003.docx]

**#AUTHOR RESPONSE TO REVIEWER 1**

**Manuscript title**: “Green City Development Principles (GCDP) Implementation Level and its Effects on Lagos Megacity Residents’ Well-being, Nigeria: A Qualitative Study”

**Manuscript ID**: PONE-D-25-25206
**Journal**: PLOS ONE
**Article type**: Original Research

**Keywords:** Green Development, City Development Principles, Urbanisation, Planning Models, Green Cities, Residents’ Well-being

**Authors’ Response**

The authors appreciate your comments, observations, suggestions, recommendations, and advice on improving the work. Thank you so much for your positive words. However, we promise to provide quality responses to all the issues. Thank you once again.

**Abstract:**
• Revise the abstract to consistently employ the past tense, ensuring alignment with the conventions of reporting completed research.
• Clarify the phrase “The findings present several obstacles” by explicitly identifying the nature of these obstacles—whether they pertain to methodological limitations, interpretive challenges, or implications for practice.
• Eliminate the word “However” from the sentence “However, the result can help...” as it introduces unnecessary contrast and does not contribute to the flow of the argument.

**Authors’ Response**

- The authors are very grateful for the detailed observations and suggestions. Regarding the abstract, consistently employing the past tense and ensuring alignment with conventional reporting have been noted and are affected in the revised manuscript.
- The phrase identified above has been clarified.
- The word “However” has been eliminated in the revised manuscript. Thank you.

**Introduction:**
• Conclude the introduction with a dedicated paragraph that clearly delineates:
o The objectives of the present study,
o Its theoretical and practical significance, and
o The scope and limitations within which the inquiry is situated.

**Authors’ Response**

Again, the authors are immensely grateful for the suggestions and advice, and we are glad to inform you that the objectives, theoretical and practical significance, scope and limitations are already included in the revised manuscript.

**Method:**
• Ensure the entire section is written in the past tense, as is standard in methodological reporting.
• Explicitly state the sampling technique employed.
• Under a subsection titled “Analysis”, detail any statistical procedures applied, including the software used if relevant.

**Authors’ Response**

Thank you for the comments and suggestions; however, the authors have ensured that the entire section is written in the past tense, the sampling technique is explicitly stated, and the analysis subsection has been enhanced and incorporated into the revised manuscript.

**Results:**
• Incorporate a table summarising the key findings derived from human participants. This will enhance clarity, facilitate comparison, and support the reader’s comprehension of the reported outcomes.

**Authors’ Response**

The authors appreciate your comments, suggestions, and recommendations, and these have been included in the revised manuscript.

**Last word**

The authors are sincerely grateful for the robust contributions that have improved this work. Thank you so much

**#AUTHOR RESPONSE TO REVIEWER 2**

**Manuscript title**: “Green City Development Principles (GCDP) Implementation Level and its Effects on Lagos Megacity Residents’ Well-being, Nigeria: A Qualitative Study”

**Manuscript ID**: PONE-D-25-25206
**Journal**: PLOS ONE
**Article type**: Original Research

**Keywords:** Green Development, City Development Principles, Urbanisation, Planning Models, Green Cities, Residents’ Well-being

**Reviewer #2:**

Since environmental issues within Lagos’ megacities pose serious health threats to urban residents, the authors aim to investigate the effect of Green City Development Plan (GCDP) implementation level as a development model to enhance residents’ well-being in Lagos megacity, Nigeria. To achieve this, the investigators used a qualitative research methodology.
Overall, the manuscript addresses an important issue that cuts across different sectors. It is also well written.

There are, however, some issues that have to be addressed to further strengthen the manuscript.
**Authors’ Response**

The authors appreciate your comments, observations, suggestions, recommendations, and advice on improving the work. Thank you so much for your positive words. However, we promise to provide quality responses to all the issues. Thank you once again.

1. The first section of the manuscript is aimed at systematically and methodologically investigating the existing literature that would properly situate the subject matter in its front burner of research. It is, however, not clear how the literatures were chosen.

**Authors’ Response**

Thank you for the comment. The authors apologise for the statement. The statement was not intended for a systematic review article. However, the use of the terms “systematically and methodologically” was intended to organise the literature review section. Regarding the process of selecting literature, they were chosen based on key phrases in the article title and the objectives.

2. It is not too clear the criteria used to choose the participants for the interviews. It will be nice for the authors to clarify this.

**Authors’ Response**

Thank you for the comments and observations. However, for clarity, a subsection ***4.2***, “sampling technique,” was created under the method and materials section to explain in more detail how participants were selected using the following criteria.

1. Experts in BEPs and HMPs
2. Lagos megacity residents living in areas with high or low GCDP implementation
3. Residents (Community leaders) actively promote sustainable practices.
4. Lagos megacity Local government officials involved in green city projects
5. Following the recommendation in qualitative research to ensure a suitable representation, the participants were picked based on:
a. lived experiences and the diversity of the experts and residents
b. age, gender, marital status, education, occupation and years of experience

The authors are grateful for the review.

3. The list of participants shows that all of them have a very high level of education. Since environmental issues tend to affect more of the low socio-economic class, I think it could have been better to include these persons as part of the participants to elicit their views. Leaving out this group of persons tend to create some bias in the findings. I think this is a limitation that should be acknowledged.

**Authors’ Response**

The authors appreciate your views, comments and suggestions. The issues in this study regarding GCDP implementation were x-rays from the perspective of the implementation level and its effects on residents’ well-being. The perspective essentially requires the opinions of operators (policymakers and governments) and expert***s***. The authors wanted to focus on the two groups and to provide opportunities for further studies of participants from low socio-economic backgrounds.

However, the authors agree with your later suggestions to acknowledge it as a limitation. The authors have captured this in the limitation section of the revised manuscript.

4. Since this is a human subject research, my thinking is that obtaining informed consent from the participants was mandatory. Except this has been waived by the ethics committee.
**Authors’ Response**

Again, thank you for your comment and observation. Yes, informed consent was issued to the participants, and it was stated in the body of the work under subsection “4.1 recruitment process”. The informed consent form was attached in ***Appendix 2.***

5. I think the discussion section can be more concise.

**Authors’ Response**

The authors are indeed very grateful for the suggestion and recommendation. We followed your advice to make the discussion section more concise in the revised manuscript. Thank you.

**Conclusion**

The authors are sincerely grateful for the robust contributions that have improved this work. Thank you so much.

**AUTHORS REPONSE TO MOST RECENT REVIEWER’S COMMENTS.**

**Manuscript title**: “Green City Development Principles (GCDP) Implementation Level and its Effects on Lagos Megacity Residents’ Well-being, Nigeria: A Qualitative Study”

**Manuscript ID**: PONE-D-25-25206
**Journal**: PLOS ONE
**Article type**: Original Research

**Keywords:** Green Development, City Development Principles, Urbanisation, Planning Models, Green Cities, Residents’ Well-being

The manuscript shows significant improvement and addresses many methodological and presentation issues.

**Authors’ Response**

The authors appreciate your comments and observations on improving the work. Thank you so much for your positive words. However, we promise to provide quality responses to all the issues. Thank you once again.

However, the following must be resolved before it can be accepted:
1. Clarify literature selection method (even briefly).

**Authors’ Response**

Thank you for the comment. A brief literature selection method has been added to the revised manuscript. Thank you.

2. Provide explicit ethical approval and consent details.

**Authors’ Response**

The authors appreciate this comment. Before data collection commenced, the application was submitted to our institutional review committee, the Covenant University Health and Research Ethics Committee (CHREC). After reviewing our eight-page protocol document submitted to CHREC, it was returned to us, stating that we need no ethical certificate because it was social research. The informed consent form was reviewed and approved by CHREC, and we attached it as a supplementary file to our original submission.

Also, we did say the data collection was from in-depth interviews with experts, but because there was no high risk, no vulnerable groups were involved, and the research did not involve life-threatening issues. Only consent forms were distributed to participants. Honestly, I do not know which other explicit ethical approval and consent details I should provide. Moreover, the research content and data are presented in the article.

3. Elaborate on the thematic analysis process (e.g., codebook development, theme refinement).

**Authors’ Response**

Thank you for your comment. We have explained the thematic analysis process for the study in the body of the work, under subsection 3.4, data analysis, ***in the materials and methods section***. However, elaborating on the thematic analysis process requires detailing each of its six steps:

**1) Familiarisation with the data**

It requires reading and re-reading the data (e.g., transcripts) to immerse yourself and identify initial meanings and patterns. Then, gain a deep understanding of the content and identify initial points of interest.

**2) Coding to identify key features**

It requires systematically tagging or labelling data segments with concise labels that capture specific ideas or concepts, breaking the data into smaller, manageable units of meaning.

**3) Searching for themes by grouping codes**

Searching for themes by grouping codes involves reviewing the codes and looking for connections and patterns to group them into potential themes. To start identifying broader topics that represent recurring patterns of meaning within the data.

**4) Reviewing potential themes to ensure coherence and clarity**

It involves critically evaluating the themes, checking for coherence within the coded extracts and ensuring each theme is distinct and meaningful in relation to the whole dataset. To refine the themes by merging similar ones, splitting overly broad themes, or discarding those that don’t contribute to the analysis.

**5) Defining and naming the finalised themes**

It requires clearly defining the essence of each theme and giving it a concise, descriptive name to establish each theme’s specific meaning and create a clear, coherent analytical framework.

6) **Writing the report with supporting data and interpretation to present the findings.**

This systematic process aims to uncover patterns and meanings relevant to the research question. It involves presenting the final analysis by writing a coherent narrative, integrating illustrative data extracts (quotes), and explaining how the themes answer the research question, which is also the research objective in its statement form. To provide a transparent, compelling, and understandable account of the data’s patterns and meanings.

4. Critically discuss the impact of participant selection bias.

**Authors’ Response**

The authors appreciate the comment. To answer your question, participant selection bias distorts research outcomes by creating a non-representative sample, thereby undermining both the internal validity (accuracy of relationships between variables) and the external validity (generalisability to the broader population) of a study. This bias leads to unreliable conclusions, inaccurate estimates of effects or risks, and loss of credibility for the research, potentially resulting in flawed decision-making.

Some of these issues were outlined as limitations to this study in the body of the work. It must be noted that the study aims to collect ***expert opinions;*** ***hence, participants were mainly experts/professionals***. However, these limitations do undermine the study’s contribution; thus, the study’s contribution under the subtheme was clearly outlined in the body of the work.

5. Provide evidence of image permissions or replace figures with CC BY 4.0 compliant ones

**Authors’ Response**

Noted, thank you for the suggestions. The corrections have been made in the revised manuscript.

.

6. Ensure all raw qualitative data are shared (e.g., via repository or supplementary files).

**Authors’ Response**

Noted and thank you for the comment. The supplementary files have been attached to the original submission.

The authors are sincerely grateful for the robust contributions that have improved this work. Thank you so much.

**#AUTHORS RESPONSE TO EDITOR COMMENTS**

**Additional Editor Comments:**
Editorial Decision: Major Revision Required

**Editor Comments to the Authors**

Thank you for submitting your manuscript. Your study addresses a relevant and timely topic: urban sustainability and public well-being in the context of the Green City Development Principles (GCDPs) in Lagos, Nigeria. The qualitative approach and local contextualization provide valuable insights. However, based on the comments from both reviewers and our editorial assessment, substantial revisions are required to meet PLOS ONE’s publication standards.

Below is a synthesis of reviewer comments and editorial recommendations that require your thorough attention:

**Authors’ Response**

The authors appreciate your comments, observations, suggestions, recommendations, and advice on improving the work. Thank you so much for your positive words. However, we promise to provide quality responses to all the issues. Thank you once again.

**1. Abstract – Tense, Clarity, and Coherence**

]• Revise to consistently employ the past tense, in accordance with reporting completed research (Reviewer 1).
• Clarify the phrase ***“several obstacles”*** by specifying the nature of these obstacles are they methodological, structural, environmental, or policy-related?
• Remove ***“However”*** from the sentence “However, the result can help...” as the contrast it introduces is unnecessary.
Action: Ensure the abstract clearly summarizes the study’s aims, design, key findings (explicitly), and implications, using concise, past-tense language.

**Authors’ Response**

Noted and thank you for the information. We have made corrections in the revised manuscript.

**2. Introduction – Focus and Structure**
• The objectives, theoretical/practical significance, and scope/limitations of the study should be more clearly and systematically delineated in the concluding paragraph of the Introduction section (Reviewer 1).
• The literature sourcing methodology is unclear (Reviewer 2). You mention using Google Scholar, Scopus, and ScienceDirect, but please clarify:
o Inclusion/exclusion criteria
o Timeframe of literature reviewed
o Any systematic or scoping technique used
Action: Enhance the last paragraph of the Introduction by clearly stating:
• Study objectives (in 1–2 concise sentences)
• Its contribution to theory and practice
• Scope and limitations
**Authors’ Response**

Noted and thank you for the information. We have made corrections in the revised manuscript. Please note that the study is not a review. Therefore, it is unnecessary to include inclusion/exclusion criteria, the Timeframe of literature reviewed, and any systematic or scoping technique used. Thank you.

**3. Methodology – Rigor and Transparency**
• Ensure the entire section is written in past tense (Reviewer 1).
• Explicitly state the sampling technique. While you mention a “purposive” approach, describe the rationale and how diversity or saturation was assured (Reviewer 2).
• The demographics in Table 1 show that participants are highly educated professionals. As pointed out by Reviewer 2, this could introduce selection bias. Please:
o Acknowledge this bias as a study limitation
o Reflect on how the exclusion of lower socio-economic groups may affect the findings’ generalizability
• Informed consent and ethics: You state ethics approval was not needed, but also mention that “a consent form approval was required.” Please clarify:
o Which institutional review board (IRB) or ethics committee reviewed or waived approval?
o Was written or verbal consent obtained? Ensure alignment with PLOS ONE’s ethics policy.
Action: Add a subsection titled “Ethical Considerations” that clearly reports IRB details, approval number (if applicable), and form of consent obtained.

**Authors’ Response**

Noted and thank you for the information. We have made corrections in the revised manuscript.

**4. Analysis – Structure and Transparency**
• Under a clear subsection titled “Data Analysis”, describe:
o Use of thematic analysis (already mentioned, but needs elaboration)
o Coding process (manual or software-assisted?)
o Number of coders and any inter-rater checks performed
Action: Add more depth to your analytical description to strengthen the rigor of the qualitative approach.
**Authors’ Response**

Noted and thank you for the information. We have made corrections in the revised manuscript.

- The coding process was manual
- The Number of coders was two (2)
- However, to assess inter-rater reliability (IRR), two independent coders were used to code the same set of data (transcripts) using the same coding scheme. After coding, the codes were compared to find areas of agreement and disagreement. Thereafter, discussions and reconciliation were carried out to conclude the process.

**5. Results – Presentation and Accessibility**
• The result section would benefit from at least one summary table presenting themes/findings derived from participant interviews (Reviewer 1). Consider a table summarizing:
o Key themes
o Number or proportion of participants who referenced the theme
o Representative quotes
Action: Add a qualitative results summary table to improve reader comprehension.
**Authors’ Response**

Noted and thank you for the information. We have made corrections in the revised manuscript.

**6. Discussion – Focus and Conciseness**
• The discussion is comprehensive but could be more concise and focused (Reviewer 2). Reduce repetition and avoid restating long background literature that was already cited in the Introduction or Results.
Action: Strengthen the discussion by:
• Summarizing how your findings answer your stated objectives
• Clearly aligning your results with the existing literature
• Highlighting practical, policy, or planning implications of GCDP implementation in Lagos
**Authors’ Response**

Noted and thank you for the information. We have made corrections in the revised manuscript.

**7. Conclusion and Limitations**
• The conclusion is generally aligned with the findings.
• However, the limitations section should be expanded to directly address:
o Participant educational skew
o Exclusion of socio-economically disadvantaged voices
o Qualitative generalizability limitations
o Potential researcher bias or recall bias during interviews
**Authors’ Response**

Noted and thank you for the information. We have made corrections in the revised manuscript.

Additional Points
• Ensure consistency in terminology (e.g., “GUDP” appears in some places and may be a typographical error for “GCDP”).
• Minor grammatical issues (e.g., article usage, verb tense) are noted throughout and should be corrected during revision.
**Authors’ Response**

Noted and thank you for the information. We have made corrections in the revised manuscript.

Summary of Required Revisions
Section Required Actions
Abstract Use past tense, clarify obstacles, remove “However”
Introduction Clearly state objectives, scope, and significance
Methodology Justify sampling, clarify consent/ethics, and write in past tense
Analysis Describe coding and analysis process more robustly
Results Add a thematic summary table
Discussion Condense and focus on interpretation and policy implications
Limitations Address participant bias, generalizability, and recall limitations
**Authors’ Response**

Noted and thank you for the information. We have made corrections in the revised manuscript.

[Note: HTML markup is below. Please do not edit.]

The authors are sincerely grateful for the additional editor’s comments on improving this work. Thank you so much.

**# Authors’ response to Journal Requirements-PLOS ONE Decision**

**PLOS ONE Decision: Revision required [PONE-D-25-25206] [EMID:1894e8cb8cae62b5]**

**Journal Requirements:**

When submitting your revision, we need you to address these additional requirements.

**Authors’ Response**

The authors appreciate your comments, observations, suggestions, recommendations, and advice on improving the work. Thank you so much for your positive words. However, we promise to provide quality responses to all the issues. Thank you once again.

1.Please ensure that your manuscript meets PLOS ONE’s style requirements, including those for file naming. The PLOS ONE style templates can be found at

<https://journals.plos.org/plosone/s/file?id=wjVg/PLOSOne_formatting_sample_main_body.pdf> and

[https://journals.plos.org/plosone/s/file?id=ba62/PLOSOne_formatting_sample_title_authors_affiliations.pdf](https://track.editorialmanager.com/CL0/https:%2F%2Fjournals.plos.org%2Fplosone%2Fs%2Ffile%3Fid=ba62%2FPLOSOne_formatting_sample_title_authors_affiliations.pdf/1/010f019818677d42-04be412e-7a5d-4fbc-a51b-ec94a195e13a-000000/WqMZfBtkJSsvqT3MA8pZW4Bx7J5fYp5u-ebQuwamA1c=220)

**Authors’ Response**

Noted and thank you for the information. We have complied with the necessary instructions.

2. We noticed you have some minor occurrence of overlapping text with the following previous publication(s), which needs to be addressed: - DOI 10.3389/frsc.2025.1506672

In your revision ensure you cite all your sources (including your own works), and quote or rephrase any duplicated text outside the methods section. Further consideration is dependent on these concerns being addressed.

**Authors’ Response**

Noted and thank you for the information. The authors have complied with the necessary instructions.

3. Thank you for stating the following in your Competing Interests section: “12975”

Please complete your Competing Interests on the online submission form to state any Competing Interests. If you have no competing interests, please state “The authors have declared that no competing interests exist.”, as detailed online in our guide for authors at <http://journals.plos.org/plosone/s/submit-now>

 This information should be included in your cover letter; we will change the online submission form on your behalf.

**Authors’ Response**

Noted and thank you for the information. We have complied with the necessary instructions.

4. Please ensure that you refer to Figures 2-8 in your text as, if accepted, production will need this reference to link the reader to the figure.

**Authors’ Response**

Noted and thank you for the information. The authors have complied with the necessary instructions.

5. Please include captions for your Supporting Information files at the end of your manuscript, and update any in-text citations to match accordingly. Please see our Supporting Information guidelines for more information: [http://journals.plos.org/plosone/s/supporting-information](http://track.editorialmanager.com/CL0/http:%2F%2Fjournals.plos.org%2Fplosone%2Fs%2Fsupporting-information/1/010f019818677d42-04be412e-7a5d-4fbc-a51b-ec94a195e13a-000000/95I2yH1kcpv32CY9dEaaL5oeHPnoeN9tXO_RWQalgL0=220).

**Authors’ Response**

Noted and thank you for the information. We have complied with the necessary instructions.

6. We note that Figures 2-4 includes an image of a participant in the study.

As per the PLOS ONE policy (<http://journals.plos.org/plosone/s/submission-guidelines#loc-human-subjects-research>) on papers that include identifying, or potentially identifying, information, the individual(s) or parent(s)/guardian(s) must be informed of the terms of the PLOS open-access (CC-BY) license and provide specific permission for publication of these details under the terms of this license.

**Authors’ Response**

Noted and thank you for the information. We have complied with the necessary instructions.

Please download the Consent Form for Publication in a PLOS Journal (<http://journals.plos.org/plosone/s/file?id=8ce6/plos-consent-form-english.pdf>). The signed consent form should not be submitted with the manuscript but should be securely filed in the individual’s case notes. Please amend the methods section and ethics statement of the manuscript to explicitly state that the patient/participant has provided consent for publication: “The individual in this manuscript has given written informed consent (as outlined in PLOS consent form) to publish these case details”.

If you are unable to obtain consent from the subject of the photograph, you will need to remove the figure and any other textual identifying information or case descriptions for this individual.

**Authors’ Response**

Noted and thank you for the information. The authors have complied with the necessary instructions.

7. We note that Figures 2-8 in your submission contain copyrighted images. All PLOS content is published under the Creative Commons Attribution License (CC BY 4.0), which means that the manuscript, images, and Supporting Information files will be freely available online, and any third party is permitted to access, download, copy, distribute, and use these materials in any way, even commercially, with proper attribution. For more information, see our copyright guidelines: <http://journals.plos.org/plosone/s/licenses-and-copyright>.

**Authors’ Response**

Noted and thank you for the information. The authors have complied with the necessary instructions.

 We require you to either (1) present written permission from the copyright holder to publish these figures specifically under the CC BY 4.0 license, or (2) remove the figures from your submission:

 1. You may seek permission from the original copyright holder of Figures 2-8 to publish the content specifically under the CC BY 4.0 license.

 We recommend that you contact the original copyright holder with the Content Permission Form (<http://journals.plos.org/plosone/s/file?id=7c09/content-permission-form.pdf>) and the following text: “I request permission for the open-access journal PLOS ONE to publish XXX under the Creative Commons Attribution License (CCAL) CC BY 4.0 (<http://creativecommons.org/licenses/by/4.0/>).

**Authors’ Response**

Noted and thank you for the information. The authors have complied with the necessary instructions.

Please be aware that this license allows unrestricted use and distribution, even commercially, by third parties. Please reply and provide explicit written permission to publish XXX under a CC BY license and complete the attached form.”

Please upload the completed Content Permission Form or other proof of granted permissions as an “Other” file with your submission.

**Authors’ Response**

Noted and thank you for the information. The authors have complied with the necessary instructions.

In the figure caption of the copyrighted figure, please include the following text: “Reprinted from [ref] under a CC BY license, with permission from [name of publisher], original copyright [original copyright year].”

**Authors’ Response**

Noted and thank you for the information. The authors have complied with the necessary instructions.

The authors have complied with the necessary instructions.

 2. If you are unable to obtain permission from the original copyright holder to publish these figures under the CC BY 4.0 license or if the copyright holder’s requirements are incompatible with the CC BY 4.0 license, please either i) remove the figure or ii) supply a replacement figure that complies with the CC BY 4.0 license. Please check copyright information on all replacement figures and update the figure caption with source information. If applicable, please specify in the figure caption text when a figure is similar but not identical to the original image and is therefore for illustrative purposes only.

**Authors’ Response**

Noted and thank you for the information. The authors have complied with the necessary instructions.

8. We note that your Data Availability Statement is currently as follows: All relevant data are within the manuscript and in Supporting Information files.

Please confirm at this time whether or not your submission contains all the raw data required to replicate the results of your study. Authors must share the “minimal data set” for their submission. PLOS defines the minimal data set to consist of the data required to replicate all study findings reported in the article, as well as related metadata and methods (<https://journals.plos.org/plosone/s/data-availability#loc-minimal-data-set-definition>).

For example, authors should submit the following data:

- The values behind the means, standard deviations and other measures reported;

- The values used to build graphs;

- The points extracted from images for analysis.

**Authors’ Response**

Yes, the authors confirm that our submission contains all the raw data required to replicate the results of your study.

Authors do not need to submit their entire data set if only a portion of the data was used in the reported study. If your submission does not contain these data, please either upload them as Supporting Information files or deposit them to a stable, public repository and provide us with the relevant URLs, DOIs, or accession numbers. For a list of recommended repositories, please see <https://journals.plos.org/plosone/s/recommended-repositories>.

**Authors’ Response**

Noted and thank you for the information. The authors have complied with the necessary instructions.

If there are ethical or legal restrictions on sharing a de-identified data set, please explain them in detail (e.g., data contain potentially sensitive information, data are owned by a third-party organization, etc.) and who has imposed them (e.g., an ethics committee). Please also provide contact information for a data access committee, ethics committee, or other institutional body to which data requests may be sent. If data are owned by a third party, please indicate how others may request data access.

**Authors’ Response**

Noted and thank you for the information. The authors have complied with the necessary instructions.

9. If the reviewer’s comments include a recommendation to cite specific previously published works, please review and evaluate these publications to determine whether they are relevant and should be cited. There is no requirement to cite these works unless the editor has indicated otherwise.

**Authors’ Response**
Noted and thank you for the comment. The authors have complied with the necessary instructions.
