# Supplementary material for: Green city development principles (GCDP) implementation level and its effects on lagos megacity residents’ well-being, Nigeria: A qualitative study
Source: PLoS One. 2026 Aug 4;21(8):e0354733. doi: 10.1371/journal.pone.0354733 (PMC13436778; doi:10.1371/journal.pone.0354733)
Supplement: Peer review history

# Peer review: Decision Letter 0

**DOI:** 10.1371/journal.pone.0354733.r001

**Authors:** Morufu Raimi

17 Jul 2025

PONE-D-25-25206

Green City Development Principles (GCDP) Implementation Level and its Effects on Lagos Megacity Residents’ Well-being, Nigeria: A Qualitative Study

PLOS ONE

Dear Dr. Ekhaese,

Thank you for submitting your manuscript to PLOS ONE. After careful consideration, we feel that it has merit but does not fully meet PLOS ONE’s publication criteria as it currently stands. Therefore, we invite you to submit a revised version of the manuscript that addresses the points raised during the review process.

Please submit your revised manuscript by Aug 31 2025 11:59PM. If you will need more time than this to complete your revisions, please reply to this message or contact the journal office at plosone@plos.org. When you’re ready to submit your revision, log on to https://www.editorialmanager.com/pone/ and select the 'Submissions Needing Revision' folder to locate your manuscript file.

Please include the following items when submitting your revised manuscript:

- A rebuttal letter that responds to each point raised by the academic editor and reviewer(s). You should upload this letter as a separate file labeled 'Response to Reviewers'.
- A marked-up copy of your manuscript that highlights changes made to the original version. You should upload this as a separate file labeled 'Revised Manuscript with Track Changes'.
- An unmarked version of your revised paper without tracked changes. You should upload this as a separate file labeled 'Manuscript'.

If you would like to make changes to your financial disclosure, please include your updated statement in your cover letter. Guidelines for resubmitting your figure files are available below the reviewer comments at the end of this letter.

If applicable, we recommend that you deposit your laboratory protocols in protocols.io to enhance the reproducibility of your results. Protocols.io assigns your protocol its own identifier (DOI) so that it can be cited independently in the future. For instructions see: https://journals.plos.org/plosone/s/submission-guidelines#loc-laboratory-protocols. Additionally, PLOS ONE offers an option for publishing peer-reviewed Lab Protocol articles, which describe protocols hosted on protocols.io. Read more information on sharing protocols at https://plos.org/protocols?utm_medium=editorial-email&utm_source=authorletters&utm_campaign=protocols.

We look forward to receiving your revised manuscript.

Kind regards,

Morufu Olalekan Raimi, Ph.D

Academic Editor

PLOS ONE

Journal Requirements:

When submitting your revision, we need you to address these additional requirements.

1.Please ensure that your manuscript meets PLOS ONE's style requirements, including those for file naming. The PLOS ONE style templates can be found at

https://journals.plos.org/plosone/s/file?id=wjVg/PLOSOne_formatting_sample_main_body.pdf and

https://journals.plos.org/plosone/s/file?id=ba62/PLOSOne_formatting_sample_title_authors_affiliations.pdf

2. We noticed you have some minor occurrence of overlapping text with the following previous publication(s), which needs to be addressed:

- DOI 10.3389/frsc.2025.1506672

In your revision ensure you cite all your sources (including your own works), and quote or rephrase any duplicated text outside the methods section. Further consideration is dependent on these concerns being addressed.

3. Thank you for stating the following in your Competing Interests section:

“12975”

Please complete your Competing Interests on the online submission form to state any Competing Interests. If you have no competing interests, please state "The authors have declared that no competing interests exist.", as detailed online in our guide for authors at http://journals.plos.org/plosone/s/submit-now

This information should be included in your cover letter; we will change the online submission form on your behalf.

4. Please ensure that you refer to Figures 2-8 in your text as, if accepted, production will need this reference to link the reader to the figure.

5. Please include captions for your Supporting Information files at the end of your manuscript, and update any in-text citations to match accordingly. Please see our Supporting Information guidelines for more information: http://journals.plos.org/plosone/s/supporting-information.

6. We note that Figures 2-4 includes an image of a participant in the study.

As per the PLOS ONE policy (http://journals.plos.org/plosone/s/submission-guidelines#loc-human-subjects-research) on papers that include identifying, or potentially identifying, information, the individual(s) or parent(s)/guardian(s) must be informed of the terms of the PLOS open-access (CC-BY) license and provide specific permission for publication of these details under the terms of this license. Please download the Consent Form for Publication in a PLOS Journal (http://journals.plos.org/plosone/s/file?id=8ce6/plos-consent-form-english.pdf). The signed consent form should not be submitted with the manuscript, but should be securely filed in the individual's case notes. Please amend the methods section and ethics statement of the manuscript to explicitly state that the patient/participant has provided consent for publication: “The individual in this manuscript has given written informed consent (as outlined in PLOS consent form) to publish these case details”.

If you are unable to obtain consent from the subject of the photograph, you will need to remove the figure and any other textual identifying information or case descriptions for this individual.

7. We note that Figures 2-8 in your submission contain copyrighted images. All PLOS content is published under the Creative Commons Attribution License (CC BY 4.0), which means that the manuscript, images, and Supporting Information files will be freely available online, and any third party is permitted to access, download, copy, distribute, and use these materials in any way, even commercially, with proper attribution. For more information, see our copyright guidelines: http://journals.plos.org/plosone/s/licenses-and-copyright.

We require you to either (1) present written permission from the copyright holder to publish these figures specifically under the CC BY 4.0 license, or (2) remove the figures from your submission:

1. You may seek permission from the original copyright holder of Figures 2-8 to publish the content specifically under the CC BY 4.0 license.

We recommend that you contact the original copyright holder with the Content Permission Form (http://journals.plos.org/plosone/s/file?id=7c09/content-permission-form.pdf) and the following text:

“I request permission for the open-access journal PLOS ONE to publish XXX under the Creative Commons Attribution License (CCAL) CC BY 4.0 (http://creativecommons.org/licenses/by/4.0/). Please be aware that this license allows unrestricted use and distribution, even commercially, by third parties. Please reply and provide explicit written permission to publish XXX under a CC BY license and complete the attached form.”

Please upload the completed Content Permission Form or other proof of granted permissions as an "Other" file with your submission.

In the figure caption of the copyrighted figure, please include the following text: “Reprinted from [ref] under a CC BY license, with permission from [name of publisher], original copyright [original copyright year].”

2. If you are unable to obtain permission from the original copyright holder to publish these figures under the CC BY 4.0 license or if the copyright holder’s requirements are incompatible with the CC BY 4.0 license, please either i) remove the figure or ii) supply a replacement figure that complies with the CC BY 4.0 license. Please check copyright information on all replacement figures and update the figure caption with source information. If applicable, please specify in the figure caption text when a figure is similar but not identical to the original image and is therefore for illustrative purposes only.

8. We note that your Data Availability Statement is currently as follows: All relevant data are within the manuscript and in Supporting Information files.

Please confirm at this time whether or not your submission contains all raw data required to replicate the results of your study. Authors must share the “minimal data set” for their submission. PLOS defines the minimal data set to consist of the data required to replicate all study findings reported in the article, as well as related metadata and methods (https://journals.plos.org/plosone/s/data-availability#loc-minimal-data-set-definition).

For example, authors should submit the following data:

- The values behind the means, standard deviations and other measures reported;

- The values used to build graphs;

- The points extracted from images for analysis.

Authors do not need to submit their entire data set if only a portion of the data was used in the reported study.

If your submission does not contain these data, please either upload them as Supporting Information files or deposit them to a stable, public repository and provide us with the relevant URLs, DOIs, or accession numbers. For a list of recommended repositories, please see https://journals.plos.org/plosone/s/recommended-repositories.

If there are ethical or legal restrictions on sharing a de-identified data set, please explain them in detail (e.g., data contain potentially sensitive information, data are owned by a third-party organization, etc.) and who has imposed them (e.g., an ethics committee). Please also provide contact information for a data access committee, ethics committee, or other institutional body to which data requests may be sent. If data are owned by a third party, please indicate how others may request data access.

9. If the reviewer comments include a recommendation to cite specific previously published works, please review and evaluate these publications to determine whether they are relevant and should be cited. There is no requirement to cite these works unless the editor has indicated otherwise.

Additional Editor Comments:

Editorial Decision: Major Revision Required

Editor Comments to the Authors

Thank you for submitting your manuscript. Your study addresses a relevant and timely topic concerning urban sustainability and public well-being in the context of Green City Development Principles (GCDPs) in Lagos, Nigeria. The qualitative approach and local contextualization provide valuable insights. However, based on the comments from both reviewers and our editorial assessment, substantial revisions are required to meet PLOS ONE’s publication standards.

Below is a synthesis of reviewer comments and editorial recommendations that require your thorough attention:

1. Abstract – Tense, Clarity, and Coherence

• Revise to consistently employ past tense, in accordance with reporting completed research (Reviewer 1).

• Clarify the phrase “several obstacles” by specifying the nature of these obstacles are they methodological, structural, environmental, or policy-related?

• Remove “However” from the sentence “However, the result can help...” as the contrast it introduces is unnecessary.

Action: Ensure the abstract clearly summarizes the study’s aims, design, key findings (explicitly), and implications, using concise, past-tense language.

2. Introduction – Focus and Structure

• The objectives, theoretical/practical significance, and scope/limitations of the study should be more clearly and systematically delineated in the concluding paragraph of the Introduction section (Reviewer 1).

• The literature sourcing methodology is unclear (Reviewer 2). You mention using Google Scholar, Scopus, and ScienceDirect, but please clarify:

o Inclusion/exclusion criteria

o Timeframe of literature reviewed

o Any systematic or scoping technique used

Action: Enhance the last paragraph of the Introduction by clearly stating:

• Study objectives (in 1–2 concise sentences)

• Its contribution to theory and practice

• Scope and limitations

3. Methodology – Rigor and Transparency

• Ensure the entire section is written in past tense (Reviewer 1).

• Explicitly state the sampling technique. While you mention a “purposive” approach, describe the rationale and how diversity or saturation was assured (Reviewer 2).

• The demographics in Table 1 show that participants are highly educated professionals. As pointed out by Reviewer 2, this could introduce selection bias. Please:

o Acknowledge this bias as a study limitation

o Reflect on how the exclusion of lower socio-economic groups may affect the findings’ generalizability

• Informed consent and ethics: You state ethics approval was not needed, but also mention that “a consent form approval was required.” Please clarify:

o Which institutional review board (IRB) or ethics committee reviewed or waived approval?

o Was written or verbal consent obtained? Ensure alignment with PLOS ONE’s ethics policy.

Action: Add a subsection titled “Ethical Considerations” that clearly reports IRB details, approval number (if applicable), and form of consent obtained.

4. Analysis – Structure and Transparency

• Under a clear subsection titled “Data Analysis”, describe:

o Use of thematic analysis (already mentioned, but needs elaboration)

o Coding process (manual or software-assisted?)

o Number of coders and any inter-rater checks performed

Action: Add more depth to your analytical description to strengthen the rigor of the qualitative approach.

5. Results – Presentation and Accessibility

• The result section would benefit from at least one summary table presenting themes/findings derived from participant interviews (Reviewer 1). Consider a table summarizing:

o Key themes

o Number or proportion of participants who referenced the theme

o Representative quotes

Action: Add a qualitative results summary table to improve reader comprehension.

6. Discussion – Focus and Conciseness

• The discussion is comprehensive but could be more concise and focused (Reviewer 2). Reduce repetition and avoid restating long background literature that was already cited in the Introduction or Results.

Action: Strengthen the discussion by:

• Summarizing how your findings answer your stated objectives

• Clearly aligning your results with the existing literature

• Highlighting practical, policy, or planning implications of GCDP implementation in Lagos

7. Conclusion and Limitations

• The conclusion is generally aligned with the findings.

• However, the limitations section should be expanded to directly address:

o Participant educational skew

o Exclusion of socio-economically disadvantaged voices

o Qualitative generalizability limitations

o Potential researcher bias or recall bias during interviews

Additional Points

• Ensure consistency in terminology (e.g., “GUDP” appears in some places and may be a typographical error for “GCDP”).

• Minor grammatical issues (e.g., article usage, verb tense) are noted throughout and should be corrected during revision.

Summary of Required Revisions

Section Required Actions

Abstract Use past tense, clarify obstacles, remove “However”

Introduction Clearly state objectives, scope, and significance

Methodology Justify sampling, clarify consent/ethics, and write in past tense

Analysis Describe coding and analysis process more robustly

Results Add a thematic summary table

Discussion Condense and focus on interpretation and policy implications

Limitations Address participant bias, generalizability, and recall limitations

[Note: HTML markup is below. Please do not edit.]

Reviewers' comments:

Reviewer's Responses to Questions

**Comments to the Author**

1. Is the manuscript technically sound, and do the data support the conclusions?

The manuscript must describe a technically sound piece of scientific research with data that supports the conclusions. Experiments must have been conducted rigorously, with appropriate controls, replication, and sample sizes. The conclusions must be drawn appropriately based on the data presented.

Reviewer #1: Yes

Reviewer #2: Yes

**********

2. Has the statistical analysis been performed appropriately and rigorously?

Reviewer #1: N/A

Reviewer #2: N/A

**********

3. Have the authors made all data underlying the findings in their manuscript fully available?

The PLOS Data policy requires authors to make all data underlying the findings described in their manuscript fully available without restriction, with rare exception (please refer to the Data Availability Statement in the manuscript PDF file). The data should be provided as part of the manuscript or its supporting information, or deposited to a public repository. For example, in addition to summary statistics, the data points behind means, medians and variance measures should be available. If there are restrictions on publicly sharing data—e.g. participant privacy or use of data from a third party—those must be specified.

Reviewer #1: Yes

Reviewer #2: Yes

**********

4. Is the manuscript presented in an intelligible fashion and written in standard English?

PLOS ONE does not copyedit accepted manuscripts, so the language in submitted articles must be clear, correct, and unambiguous. Any typographical or grammatical errors should be corrected at revision, so please note any specific errors here.

Reviewer #1: Yes

Reviewer #2: Yes

**********

5. Review Comments to the Author

Please use the space provided to explain your answers to the questions above. You may also include additional comments for the author, including concerns about dual publication, research ethics, or publication ethics. (Please upload your review as an attachment if it exceeds 20,000 characters)

Reviewer #1: Abstract:

• Revise the abstract to consistently employ the past tense, ensuring alignment with the conventions of reporting completed research.

• Clarify the phrase "The findings present several obstacles" by explicitly identifying the nature of these obstacles—whether they pertain to methodological limitations, interpretive challenges, or implications for practice.

• Eliminate the word "However" from the sentence "However, the result can help..." as it introduces unnecessary contrast and does not contribute to the flow of the argument.

Introduction:

• Conclude the introduction with a dedicated paragraph that clearly delineates:

o The objectives of the present study,

o Its theoretical and practical significance, and

o The scope and limitations within which the inquiry is situated.

Method:

• Ensure the entire section is written in the past tense, as is standard in methodological reporting.

• Explicitly state the sampling technique employed.

• Under a subsection titled "Analysis", detail any statistical procedures applied including the software used if relevant.

Results:

• Incorporate a table summarizing the key findings derived from human participants. This will enhance clarity, facilitate comparison, and support the reader’s comprehension of the reported outcomes.

Reviewer #2: Since environmental issues within Lagos’ megacities pose serious health threats to urban residents, the authors aim to investigate the effect of Green City Development Plan (GCDP’s) implementation level as a development

model to enhance residents’ well-being in Lagos megacity, Nigeria. To achieve this, the investigators used a qualitative research methodology.

Overall, the manuscript addresses an important issue that cuts across different sectors. It is also well written.

There are however some issues that have to be addressed to further strengthen the manuscript.

1.The first section of the manuscript is aimed at systematically and methodologically investigating the existing literature that would properly situate the subject matter in its front burner of research. It is however not clear how the literatures were chosen.

2.It is not too clear the criteria used to choose the participants for the interviews. It will be nice for the authors to clarify this.

3. The list of participants shows that all of them have very high level of education. Since, environmental issues tend to affect more of the low socio-economic class, I think it could have been better to include these persons as part of the participants to elicit their views. Leaving out this group of persons tend to create some bias in the findings. I think this is a limitation that should be acknowledged.

4. Since this is a human subject research, my thinking is that obtaining informed consent from the participants was mandatory. Except this has been waved by the ethics committee.

5. I think the discussion section can be more concise.

**********

6. PLOS authors have the option to publish the peer review history of their article (what does this mean?). If published, this will include your full peer review and any attached files.

If you choose “no”, your identity will remain anonymous but your review may still be made public.

**Do you want your identity to be public for this peer review?**  For information about this choice, including consent withdrawal, please see our Privacy Policy.

Reviewer #1: No

Reviewer #2: No

**********

[NOTE: If reviewer comments were submitted as an attachment file, they will be attached to this email and accessible via the submission site. Please log into your account, locate the manuscript record, and check for the action link "View Attachments". If this link does not appear, there are no attachment files.]

While revising your submission, please upload your figure files to the Preflight Analysis and Conversion Engine (PACE) digital diagnostic tool, https://pacev2.apexcovantage.com/. PACE helps ensure that figures meet PLOS requirements. To use PACE, you must first register as a user. Registration is free. Then, login and navigate to the UPLOAD tab, where you will find detailed instructions on how to use the tool. If you encounter any issues or have any questions when using PACE, please email PLOS at figures@plos.org. Please note that Supporting Information files do not need this step.

# Author comment: Author response to Decision Letter 1

**DOI:** 10.1371/journal.pone.0354733.r002

5 Sep 2025

#Author Response to Reviewer 1

Manuscript title: “Green City Development Principles (GCDP) Implementation Level and its Effects on Lagos Megacity Residents’ Well-being, Nigeria: A Qualitative Study”

Manuscript ID: PONE-D-25-25206

Journal: PLOS ONE

Article type: Original Research

Keywords: Green Development, City Development Principles, Urbanisation, Planning Models, Green Cities, Residents’ Well-being

Authors’ Response

The authors appreciate your comments, observations, suggestions, recommendations, and advice on improving the work. Thank you so much for your positive words. However, we promise to provide quality responses to all the issues. Thank you once again.

Abstract:

• Revise the abstract to consistently employ the past tense, ensuring alignment with the conventions of reporting completed research.

• Clarify the phrase “The findings present several obstacles” by explicitly identifying the nature of these obstacles—whether they pertain to methodological limitations, interpretive challenges, or implications for practice.

• Eliminate the word “However” from the sentence “However, the result can help...” as it introduces unnecessary contrast and does not contribute to the flow of the argument.

Authors’ Response

• The authors are very grateful for the detailed observations and suggestions. Regarding the abstract, consistently employing the past tense and ensuring alignment with conventional reporting have been noted and are affected in the revised manuscript.

• The phrase identified above has been clarified.

• The word “However” has been eliminated in the revised manuscript. Thank you.

Introduction:

• Conclude the introduction with a dedicated paragraph that clearly delineates:

o The objectives of the present study,

o Its theoretical and practical significance, and

o The scope and limitations within which the inquiry is situated.

Authors’ Response

Again, the authors are immensely grateful for the suggestions and advice, and we are glad to inform you that the objectives, theoretical and practical significance, scope and limitations are already included in the revised manuscript.

Method:

• Ensure the entire section is written in the past tense, as is standard in methodological reporting.

• Explicitly state the sampling technique employed.

• Under a subsection titled “Analysis”, detail any statistical procedures applied, including the software used if relevant.

Authors’ Response

Thank you for the comments and suggestions; however, the authors have ensured that the entire section is written in the past tense, the sampling technique is explicitly stated, and the analysis subsection has been enhanced and incorporated into the revised manuscript.

Results:

• Incorporate a table summarising the key findings derived from human participants. This will enhance clarity, facilitate comparison, and support the reader’s comprehension of the reported outcomes.

Authors’ Response

The authors appreciate your comments, suggestions, and recommendations, and these have been included in the revised manuscript.

Last word

The authors are sincerely grateful for the robust contributions to improving this work. Thank you so much

#Author Response to Reviewer 2

Manuscript title: “Green City Development Principles (GCDP) Implementation Level and its Effects on Lagos Megacity Residents’ Well-being, Nigeria: A Qualitative Study”

Manuscript ID: PONE-D-25-25206

Journal: PLOS ONE

Article type: Original Research

Keywords: Green Development, City Development Principles, Urbanisation, Planning Models, Green Cities, Residents’ Well-being

Reviewer #2:

Since environmental issues within Lagos’ megacities pose serious health threats to urban residents, the authors aim to investigate the effect of Green City Development Plan (GCDP) implementation level as a development model to enhance residents’ well-being in Lagos megacity, Nigeria. To achieve this, the investigators used a qualitative research methodology.

Overall, the manuscript addresses an important issue that cuts across different sectors. It is also well written.

There are, however, some issues that have to be addressed to further strengthen the manuscript.

Authors’ Response

The authors appreciate your comments, observations, suggestions, recommendations, and advice on improving the work. Thank you so much for your positive words. However, we promise to provide quality responses to all the issues. Thank you once again.

1. The first section of the manuscript is aimed at systematically and methodologically investigating the existing literature that would properly situate the subject matter in its front burner of research. It is, however, not clear how the literatures were chosen.

Authors’ Response

Thank you for the comment. The authors apologise for the statement. The statement was not used with a systematic review article in mind. However, the use of the terms “systematically and methodologically” was to position the literature review section in an organised manner. Regarding the process of choosing literature, they were chosen using the key phrases in the article title and the objectives.

2. It is not too clear the criteria used to choose the participants for the interviews. It will be nice for the authors to clarify this.

Authors’ Response

Thank you for the comments and observations. However, for clarity, a subsection 4.2 “sampling technique” was created under the method and material section to explain further how the participants were chosen using the following criteria.

1. Experts in BEPs and HMPs

2. Lagos megacity residents living in areas with high or low GCDP implementation

3. Residents (Community leaders) actively promote sustainable practices.

4. Lagos megacity Local government officials involved in green city projects

5. Following the recommendation in qualitative research to ensure a suitable representation, the participants were picked based on:

a. lived experiences and the diversity of the experts and residents

b. age, gender, marital status, education, occupation and years of experience

The authors are grateful for the review.

3. The list of participants shows that all of them have a very high level of education. Since environmental issues tend to affect more of the low socio-economic class, I think it could have been better to include these persons as part of the participants to elicit their views. Leaving out this group of persons tend to create some bias in the findings. I think this is a limitation that should be acknowledged.

Authors’ Response

The authors appreciate your views, comments and suggestions. The issues of GCDP implementation in this study were x-rays from the perspective of the level of implementation and its effects on residents’ well-being. The perspective essentially requires the opinion of operators (policymakers and governments) and experts’ opinions. The authors wanted to focus on the two groups and allow opportunities for further studies on the participants from the low socio-economic class.

However, the authors agree with your later suggestions to acknowledge it as a limitation. The authors have captured this in the limitation section of the revised manuscript.

4. Since this is a human subject research, my thinking is that obtaining informed consent from the participants was mandatory. Except this has been waived by the ethics committee.

Authors’ Response

Again, thank you for your comment and observation. Yes, informed consent was issued to the participants, and it was stated in the body of the work under subsection “4.1 recruitment process”. The informed consent form was attached in Appendix 2.

5. I think the discussion section can be more concise.

Authors’ Response

The authors are indeed very grateful for the suggestion and recommendation. We followed your advice to make the discussion section more concise in the revised manuscript. Thank you.

Conclusion

The authors are sincerely grateful for the robust contributions to improving this work. Thank you so much.

#Authors response to Editor Comments

Additional Editor Comments:

Editorial Decision: Major Revision Required

Editor Comments to the Authors

Thank you for submitting your manuscript. Your study addresses a relevant and timely topic concerning urban sustainability and public well-being in the context of Green City Development Principles (GCDPs) in Lagos, Nigeria. The qualitative approach and local contextualization provide valuable insights. However, based on the comments from both reviewers and our editorial assessment, substantial revisions are required to meet PLOS ONE’s publication standards.

Below is a synthesis of reviewer comments and editorial recommendations that require your thorough attention:

Authors’ Response

The authors appreciate your comments, observations, suggestions, recommendations, and advice on improving the work. Thank you so much for your positive words. However, we promise to provide quality responses to all the issues. Thank you once again.

1. Abstract – Tense, Clarity, and Coherence

• Revise to consistently employ past tense, in accordance with reporting completed research (Reviewer 1).

• Clarify the phrase “several obstacles” by specifying the nature of these obstacles are they methodological, structural, environmental, or policy-related?

• Remove “However” from the sentence “However, the result can help...” as the contrast it introduces is unnecessary.

Action: Ensure the abstract clearly summarizes the study’s aims, design, key findings (explicitly), and implications, using concise, past-tense language.

Authors’ Response

Noted and thank you for the information. We have made corrections in the revised manuscript.

2. Introduction – Focus and Structure

• The objectives, theoretical/practical significance, and scope/limitations of the study should be more clearly and systematically delineated in the concluding paragraph of the Introduction section (Reviewer 1).

• The literature sourcing methodology is unclear (Reviewer 2). You mention using Google Scholar, Scopus, and ScienceDirect, but please clarify:

o Inclusion/exclusion criteria

o Timeframe of literature reviewed

o Any systematic or scoping technique used

Action: Enhance the last paragraph of the Introduction by clearly stating:

• Study objectives (in 1–2 concise sentences)

• Its contribution to theory and practice

• Scope and limitations

Authors’ Response

Noted and thank you for the information. We have made corrections in the revised manuscript. Please note that the study is not a review. Therefore, it is unnecessary to include inclusion/exclusion criteria, the Timeframe of literature reviewed, and any systematic or scoping technique used. Thank you.

3. Methodology – Rigor and Transparency

• Ensure the entire section is written in past tense (Reviewer 1).

• Explicitly state the sampling technique. While you mention a “purposive” approach, describe the rationale and how diversity or saturation was assured (Reviewer 2).

• The demographics in Table 1 show that participants are highly educated professionals. As pointed out by Reviewer 2, this could introduce selection bias. Please:

o Acknowledge this bias as a study limitation

o Reflect on how the exclusion of lower socio-economic groups may affect the findings’ generalizability

• Informed consent and ethics: You state ethics approval was not needed, but also mention that “a consent form approval was required.” Please clarify:

o Which institutional review board (IRB) or ethics committee reviewed or waived approval?

o Was written or verbal consent obtained? Ensure alignment with PLOS ONE’s ethics policy.

Action: Add a subsection titled “Ethical Considerations” that clearly reports IRB details, approval number (if applicable), and form of consent obtained.

Authors’ Response

Noted and thank you for the information. We have made corrections in the revised manuscript.

4. Analysis – Structure and Transparency

• Under a clear subsection titled “Data Analysis”, describe:

o Use of thematic analysis (already mentioned, but needs elaboration)

o Coding process (manual or software-assisted?)

o Number of coders and any inter-rater checks performed

Action: Add more depth to your analytical description to strengthen the rigor of the qualitative approach.

Authors’ Response

Noted and thank you for the information. We have made corrections in the revised manuscript.

• The coding process was manual

• The Number of coders was two (2)

• However, to perform an inter-rater reliability (IRR) check, two independent coders were used to code the same set of data (transcripts) using the same coding scheme. After coding, the codes were compared to find areas of agreement and disagreement. Thereafter, discussions and reconciliation were carried out to conclude the process.

5. Results – Presentation and Accessibility

• The result section would benefit from at least one summary table presenting themes/findings derived from participant interviews (Reviewer 1). Consider a table summarizing:

o Key themes

o Number or proportion of participants who referenced the theme

o Representative quotes

Action: Add a qualitative results summary table to improve reader comprehension.

Authors’ Response

Noted and thank you for the information. We have made corrections in the revised manuscript.

6. Discussion – Focus and Conciseness

• The discussion is comprehensive but could be more concise and focused (Reviewer 2). Reduce repetition and avoid restating long background literature that was already cited in the Introduction or Results.

Action: Strengthen the discussion by:

• Summarizing how your findings answer your stated objectives

• Clearly aligning your results with the existing literature

• Highlighting practical, policy, or planning implications of GCDP implementation in Lagos

Authors’ Response

Noted and thank you for the information. We have made corrections in the revised manuscript.

7. Conclusion and Limitations

• The conclusion is generally aligned with the findings.

• However, the limitations section should be expanded to directly address:

o Participant educational skew

o Exclusion of socio-economically disadvantaged voices

o Qualitative generalizability limitations

o Potential researcher bias or recall bias during interviews

Authors’ Response

Noted and thank you for the information. We have made corrections in the revised manuscript.

Additional Points

• Ensure consistency in terminology (e.g., “GUDP” appears in some places and may be a typographical error for “GCDP”).

• Minor grammatical issues (e.g., article usage, verb tense) are noted throughout and should be corrected during revision.

Authors’ Response

Noted and thank you for the information. We have made corrections in the revised manuscript.

Summary of Required Revisions

Section Required Actions

Abstract Use past tense, clarify obstacles, remove “However”

Introduction Clearly state objectives, scope, and significance

Methodology Justify sampling, clarify consent/ethics, and write in past tense

Analysis Describe coding and analysis process more robustly

Results Add a thematic summary table

Discussion Condense and focus on interpretation and policy implications

Limitations Address participant bias, generalizability, and recall limitations

Authors’ Response

Noted and thank you for the information. We have made corrections in the revised manuscript.

[Note: HTML markup is below. Please do not edit.]

The authors are sincerely grateful for the additional editor’s comments on improving this work. Thank you so much.

#Authors' response to Journal Requirements-PLOS ONE Decision

PLOS ONE Decision: Revision required [PONE-D-25-25206] [EMID:1894e8cb8cae62b5]

Journal Requirements:

When submitting your revision, we need you to address these additional requirements.

Authors’ Response

The authors appreciate your comments, observations, suggestions, recommendations, and advice on improving the work. Thank you so much for your positive words. However, we promise to provide quality responses to all the issues. Thank you once again.

1.Please ensure that your manuscript meets PLOS ONE’s style requirements, including those for file naming. The PLOS ONE style templates can be found

Attachment Submitted filename: Authors response to Journal Requirements-PLOS ONE Decision.docx

# Editor report: Decision Letter 1

**DOI:** 10.1371/journal.pone.0354733.r003

**Authors:** Morufu Raimi

22 Sep 2025

PONE-D-25-25206R1

Green City Development Principles (GCDP) Implementation Level and its Effects on Lagos Megacity Residents’ Well-being, Nigeria: A Qualitative Study

PLOS ONE

Dear Dr. Ekhaese,

Thank you for submitting your manuscript to PLOS ONE. After careful consideration, we feel that it has merit but does not fully meet PLOS ONE’s publication criteria as it currently stands. Therefore, we invite you to submit a revised version of the manuscript that addresses the points raised during the review process.

Please submit your revised manuscript by Nov 06 2025 11:59PM. If you will need more time than this to complete your revisions, please reply to this message or contact the journal office at plosone@plos.org. When you're ready to submit your revision, log on to https://www.editorialmanager.com/pone/ and select the 'Submissions Needing Revision' folder to locate your manuscript file.

Please include the following items when submitting your revised manuscript:

- A rebuttal letter that responds to each point raised by the academic editor and reviewer(s). You should upload this letter as a separate file labeled 'Response to Reviewers'.
- A marked-up copy of your manuscript that highlights changes made to the original version. You should upload this as a separate file labeled 'Revised Manuscript with Track Changes'.
- An unmarked version of your revised paper without tracked changes. You should upload this as a separate file labeled 'Manuscript'.

If you would like to make changes to your financial disclosure, please include your updated statement in your cover letter. Guidelines for resubmitting your figure files are available below the reviewer comments at the end of this letter.

If applicable, we recommend that you deposit your laboratory protocols in protocols.io to enhance the reproducibility of your results. Protocols.io assigns your protocol its own identifier (DOI) so that it can be cited independently in the future. For instructions see: https://journals.plos.org/plosone/s/submission-guidelines#loc-laboratory-protocols. Additionally, PLOS ONE offers an option for publishing peer-reviewed Lab Protocol articles, which describe protocols hosted on protocols.io. Read more information on sharing protocols at https://plos.org/protocols?utm_medium=editorial-email&utm_source=authorletters&utm_campaign=protocols.

We look forward to receiving your revised manuscript.

Kind regards,

Morufu Olalekan Raimi, Ph.D

Academic Editor

PLOS ONE

Journal Requirements:

If the reviewer comments include a recommendation to cite specific previously published works, please review and evaluate these publications to determine whether they are relevant and should be cited. There is no requirement to cite these works unless the editor has indicated otherwise.

**Additional Editor Comments:**

PONE-D-25-25206

Overall Assessment

The authors have made a substantial effort to address the majority of the reviewers' and editors' comments. However, not all concerns have been fully resolved, and several critical issues remain that prevent the manuscript from meeting PLOS ONE’s publication standards in its current form.

Detailed Evaluation of Responses

Fully Addressed Comments:

1. Abstract:

o Past tense applied consistently.

o The phrase “several obstacles” has been clarified to refer to structural, environmental, and policy-related barriers.

o The word “However” has been removed.

2. Introduction:

o A dedicated concluding paragraph now clearly states the study's objectives, theoretical and practical significance, and scope/limitations.

3. Sampling Technique:

o Explicitly described under “3.2 Sampling Technique” (purposive, snowball, convenience).

4. Informed Consent:

o Clarified under “Ethical Considerations” and “3.1 Recruitment Process”.

5. Table of Key Findings:

o Added as Table 3, summarizing findings from human participants.

6. Discussion Section:

o Made more concise and focused on interpretation and policy implications.

7. Limitations Section:

o Expanded to address participant bias, generalizability, and recall bias.

8. Terminology Consistency:

o “GUDP” typo corrected to “GCDP” throughout.

Partially Addressed or Unresolved Issues:

1. Literature Sourcing Methodology:

o The authors state that inclusion/exclusion criteria and systematic techniques are “unnecessary” because the study is not a review.

o This is insufficient. Even in a non-review paper, the method of literature selection must be transparent to ensure scholarly rigor. The authors should briefly describe how sources were identified and selected (e.g., keywords, databases, time frame).

2. Ethical Approval Details:

o The authors mention that “ethical approval was not needed,” but also refer to an ethics committee (CUHREC) and consent forms.

o This is contradictory. They must clearly state:

• Which ethics committee waived the requirement (if applicable)

• Whether written or verbal consent was obtained

• How consent was documented

3. Coding and Analysis Process:

o The authors state that coding was manual and involved two coders with inter-rater reliability checks.

o However, no detail is provided on how themes were developed, how disagreements were resolved, or whether software was used for analysis (e.g., NVivo, MAXQDA). This lacks depth for a qualitative study.

4. Selection Bias and Generalizability:

o While the limitation is acknowledged, the authors do not adequately reflect on how the exclusion of lower socio-economic groups affects the findings. This should be discussed more critically in the limitations section.

5. Copyright and Image Permissions:

o The authors state they have “complied,” but no evidence is provided that they obtained CC BY 4.0 permissions for Figures 2-8 or replaced them with open-license alternatives.

o This must be confirmed before publication.

6. Data Availability:

o The authors confirm that all data are included, but no minimal data set (e.g., interview transcripts, coded data) is provided in the submission.

o PLOS ONE requires that qualitative data be made available in a de-identified form or deposited in a repository.

Recommendation

Major Revision is Still Required.

The manuscript shows significant improvement and addresses many methodological and presentation issues. However, the following must be resolved before it can be accepted:

1. Clarify literature selection method (even briefly).

2. Provide explicit ethical approval and consent details.

3. Elaborate on the thematic analysis process (e.g., codebook development, theme refinement).

4. Critically discuss the impact of participant selection bias.

5. Provide evidence of image permissions or replace figures with CC BY 4.0 compliant ones.

6. Ensure all raw qualitative data are shared (e.g., via repository or supplementary files).

Final Decision

Revise and Resubmit: The manuscript is not yet acceptable for publication but may be reconsidered after these specific issues are addressed.

Let me know if you would like a marked-up version of the manuscript or a draft response to the authors.

Sincerely,

Dr. Morufu Olalekan RAIMI,

BSc, (Geography. and Environmental Management), Diploma. (Environmental Health), M.Sc. Environmental Health Management), M.Phil. (Environmental Health Science), P.hD (Environmental Health Science), MNES, REHO, LEHO, FAIWMES

Environmental Health Consultant/Lecturer at Federal University Otuoke, Bayelsa State. Nigeria.

Environmental Health Consultant to United Nations Economic Commission for Europe (UNECE) Expert Group on Resources Management (EGRM). Geneva, Switzerland.

Research Consultant to Bayelsa State Primary Health Care Board.

Former Technical Adviser to the Executive Secretary, Bayelsa State Primary Health Care Board.

Former Director, Advocacy, Communication and Social Mobilization, Bayelsa State Primary Health Care Board.

Program Manager, Centre for Niger Delta Studies and Sustainability (CNDSS), Federal University Otuoke, Bayelsa State.

Deputy Director, Niger Delta Institute for Emerging and Re-emerging Infectious Diseases (NDIERID), Federal University Otuoke, Bayelsa State.

Reviewer to National Science Foundation (NSF) Graduate Research Fellowship Program (GRFP)

https://publons.com/a/1479339/

https://ssrn.com/author=2891311

ORCID iD: https://orcid.org/0000-0001-5042-6729

Web of Science Researcher ID: https://publons.com/a/1479339/

Website: https://ssrn.com/author=2891311; https://www.growkudos.com/profile/morufu_raimi; https://sciprofiles.com/profile/Morufuolalekanraimi; https://livedna.org/234.27529

https://scholar.google.com/citations?user=nRBW82AAAAAJ&hl=en.

https://theconversation.com/profiles/morufu-olalekan-raimi-1520774

[Note: HTML markup is below. Please do not edit.]

[NOTE: If reviewer comments were submitted as an attachment file, they will be attached to this email and accessible via the submission site. Please log into your account, locate the manuscript record, and check for the action link "View Attachments". If this link does not appear, there are no attachment files.]

While revising your submission, please upload your figure files to the Preflight Analysis and Conversion Engine (PACE) digital diagnostic tool, https://pacev2.apexcovantage.com/. PACE helps ensure that figures meet PLOS requirements. To use PACE, you must first register as a user. Registration is free. Then, login and navigate to the UPLOAD tab, where you will find detailed instructions on how to use the tool. If you encounter any issues or have any questions when using PACE, please email PLOS at figures@plos.org. Please note that Supporting Information files do not need this step.

# Author comment: Author response to Decision Letter 2

**DOI:** 10.1371/journal.pone.0354733.r004

26 May 2026

Manuscript title: “Green City Development Principles (GCDP) Implementation Level and its Effects on Lagos Megacity Residents’ Well-being, Nigeria: A Qualitative Study”

Manuscript ID: PONE-D-25-25206

Journal: PLOS ONE

Article type: Original Research

Keywords: Green Development, City Development Principles, Urbanisation, Planning Models, Green Cities, Residents’ Well-being

The manuscript shows significant improvement and addresses many methodological and presentation issues.

Authors’ Response

The authors appreciate your comments and observations on the improvement of the work. Thank you so much for your positive words. However, we promise to provide quality responses to all the issues. Thank you once again.

However, the following must be resolved before it can be accepted:

1. Clarify literature selection method (even briefly).

Authors’ Response

Thank you for the comment. A brief literature selection method has been added to the revised manuscript. Thank you.

2. Provide explicit ethical approval and consent details.

Authors’ Response

The authors appreciate this comment. Before the data collection commenced, the application was sent to our institutional review committee - Covenant University Health and Research Ethics Committee (CHREC). After reviewing our eight-page protocol document submitted to CHREC, it was returned to us, stating that we need no ethical certificate because it was social research. So, the informed consent form was reviewed and approved by CHREC, which we attached as supplementary files in our original submission.

Also, we did say the data collection was from in-depth interviews with experts, but because there was no high risk, no vulnerable group was involved, and the research did not require life-threatening issues. Only consent forms were distributed to participants. Honestly, I do not know which other explicit ethical approval and consent details I should provide. Moreover, the research content and data are presented in the article.

3. Elaborate on the thematic analysis process (e.g., codebook development, theme refinement).

Authors’ Response

Thank you for your comment. We have explained the thematic analysis process for the study in the body of the work under subsection 3.4 data analysis in the materials and methods section. However, elaborating on the thematic analysis process requires detailing each of its six steps:

1) Familiarisation with the data

It requires reading and re-reading the data (e.g., transcripts) to immerse yourself and identify initial meanings and patterns. Then, gain a deep understanding of the content and identify initial points of interest.

2) Coding to identify key features

It requires systematically tagging or labelling segments of the data with concise labels that capture specific ideas or concepts to break down the data into smaller, manageable units of meaning.

3) Searching for themes by grouping codes

Searching for themes by grouping codes involves reviewing the codes and looking for connections and patterns to group them into potential themes. To start identifying broader topics that represent recurring patterns of meaning within the data.

4) Reviewing potential themes to ensure coherence and clarity

It involves critically evaluating the themes, checking for coherence within the coded extracts and ensuring each theme is distinct and meaningful in relation to the whole dataset. To refine the themes by merging similar ones, splitting overly broad themes, or discarding those that don’t contribute to the analysis.

5) Defining and naming the finalised themes

It requires clearly defining the essence of each theme and giving it a concise, descriptive name to ensure each theme’s specific meaning is established and to create a clear, coherent analytical framework.

6) Writing the report with supporting data and interpretation to present the findings.

This systematic process aims to uncover patterns and meanings relevant to the research question. It involves presenting the final analysis by writing a coherent narrative, integrating illustrative data extracts (quotes), and explaining how the themes answer the research question, which is also the research objective in its statement form. To provide a transparent, compelling, and understandable account of the data’s patterns and meanings.

4. Critically discuss the impact of participant selection bias.

Authors’ Response

The authors appreciate the comment. To answer your question, participant selection bias distorts research outcomes by creating a non-representative sample, which undermines both the internal validity (accuracy of relationships between variables) and external validity (generalisability to the broader population) of a study. This bias leads to unreliable conclusions, inaccurate estimates of effects or risks, and loss of credibility for the research, potentially resulting in flawed decision-making.

Some of these issues were outlined as limitations to this study in the body of the work. It must be stated here that the study is set to collect data from expert opinions; hence, participants were mainly experts/professionals. However, these limitations do undermine the contribution of the study; thus, the contribution of the study under the subtheme contribution of the study was clearly outlined in the body of the work.

5. Provide evidence of image permissions or replace figures with CC BY 4.0 compliant ones

Authors’ Response

Noted, thank you for the suggestions. The corrections have been made in the revised manuscript.

6. Ensure all raw qualitative data are shared (e.g., via repository or supplementary files).

Authors’ Response

Noted and thank you for the comment. The supplementary files have been attached to the original submission.

The authors are sincerely grateful for the robust contributions to improving this work. Thank you so much.

Attachment Submitted filename: Response to Reviewers letter.docx

# Editor report: Decision Letter 2

**DOI:** 10.1371/journal.pone.0354733.r005

**Authors:** Morufu Raimi

1 Jun 2026

PONE-D-25-25206R2

Green City Development Principles (GCDP) Implementation Level and its Effects on Lagos Megacity Residents’ Well-being, Nigeria: A Qualitative Study

PLOS One

Dear Dr. Ekhaese,

Thank you for submitting your manuscript to PLOS ONE. After careful consideration, we feel that it has merit but does not fully meet PLOS ONE’s publication criteria as it currently stands. Therefore, we invite you to submit a revised version of the manuscript that addresses the points raised during the review process.

Please submit your revised manuscript by Jul 16 2026 11:59PM. If you will need more time than this to complete your revisions, please reply to this message or contact the journal office at plosone@plos.org. When you're ready to submit your revision, log on to https://www.editorialmanager.com/pone/ and select the 'Submissions Needing Revision' folder to locate your manuscript file.

Please include the following items when submitting your revised manuscript:

- A letter that responds to each point raised by the academic editor and reviewer(s). You should upload this letter as a separate file labeled 'Response to Reviewers'.
- A marked-up copy of your manuscript that highlights changes made to the original version. You should upload this as a separate file labeled 'Revised Manuscript with Track Changes'.
- An unmarked version of your revised paper without tracked changes. You should upload this as a separate file labeled 'Manuscript'.

If you would like to make changes to your financial disclosure, please include your updated statement in your cover letter. Guidelines for resubmitting your figure files are available below the reviewer comments at the end of this letter.

If applicable, we recommend that you deposit your laboratory protocols in protocols.io to enhance the reproducibility of your results. Protocols.io assigns your protocol its own identifier (DOI) so that it can be cited independently in the future. For instructions see: https://journals.plos.org/plosone/s/submission-guidelines#loc-laboratory-protocols. Additionally, PLOS ONE offers an option for publishing peer-reviewed Lab Protocol articles, which describe protocols hosted on protocols.io. Read more information on sharing protocols at https://plos.org/protocols?utm_medium=editorial-email&utm_source=authorletters&utm_campaign=protocols.

As the corresponding author, your ORCID iD is verified in the submission system and will appear in the published article. PLOS supports the use of ORCID, and we encourage all coauthors to register for an ORCID iD and use it as well. Please encourage your coauthors to verify their ORCID iD within the submission system before final acceptance, as unverified ORCID iDs will not appear in the published article. *Only*  the individual author can complete the verification step; PLOS staff *cannot*  verify ORCID iDs on behalf of authors.

We look forward to receiving your revised manuscript.

Kind regards,

Morufu Olalekan Raimi, Ph.D

Academic Editor

PLOS One

Journal Requirements:

If the reviewer comments include a recommendation to cite specific previously published works, please review and evaluate these publications to determine whether they are relevant and should be cited. There is no requirement to cite these works unless the editor has indicated otherwise.

Please review your reference list to ensure that it is complete and correct. If you have cited papers that have been retracted, please include the rationale for doing so in the manuscript text, or remove these references and replace them with relevant current references. Any changes to the reference list should be mentioned in the rebuttal letter that accompanies your revised manuscript. If you need to cite a retracted article, indicate the article’s retracted status in the References list and also include a citation and full reference for the retraction notice.

Additional Editor Comments:

PLOS ONE Editorial Decision

Manuscript ID: PONE-D-25-25206R1

Title: Green City Development Principles (GCDP) Implementation Level and its Effects on Lagos Megacity Residents’ Well-being, Nigeria: A Qualitative Study

Corresponding Author: Eghosa N. Ekhaese

Decision: Minor Revision (conditional acceptance)

OVERALL ASSESSMENT

The authors have responded substantively to the required revisions from the previous round. I acknowledge that they have:

• Clarified the literature selection method briefly.

• Added explicit ethical consideration details (CHREC review, written informed consent).

• Elaborated on the six-step thematic analysis process.

• Expanded the limitations section to address participant selection bias.

• Confirmed compliance with image permissions and data sharing.

The manuscript is now substantially improved in methodological transparency and scholarly rigor. However, three discrete issues remain unresolved. These are minor and can be corrected without re-review.

UNRESOLVED ISSUES (MINOR, REQUIRING FINAL CORRECTIONS)

1. Ethical approval statement remains internally inconsistent

In the revised manuscript (pages 66-67), the authors state: “the application was sent to CHREC… it was returned to us that we need no ethical certificate because it was social research.” Then they write: “ethical approval was not needed because there was no high risk, no vulnerable group involved.”

This is problematic. A formal waiver of ethical review is still an ethics determination. The correct phrasing should be: “CHREC reviewed the protocol and issued a formal waiver of full ethical approval, determining that the study presented no more than minimal risk and did not involve vulnerable populations. Written informed consent was obtained from all participants.”

Required action: Revise the Ethical Considerations subsection to explicitly state that a waiver was granted, not that approval was “not needed.” Provide the date of the CHREC waiver determination if available.

2. Thematic analysis – no evidence of inter-rater reliability process in the manuscript

In the author response, they mention two coders and an IRR check. However, the revised manuscript (page 67) does not include this detail. It only describes the six-step process generically.

Required action: Add one sentence to the Data Analysis subsection stating: “Two independent coders manually coded the transcripts. Inter-rater reliability was assessed by comparing codes; disagreements were resolved through discussion and reconciliation.”

3. Raw qualitative data not verifiably shared

The authors state that supplementary files have been attached. However, in the reviewed submission files, no de-identified interview transcripts or codebook are present. PLOS ONE requires the minimal data set (or a clear explanation of restrictions).

Required action: Either:

• Upload de-identified transcripts (or a representative sample) as supporting information, or

• Deposit them in a public repository (e.g., Figshare, Zenodo) and provide the DOI, or

• Provide a written statement from CHREC prohibiting sharing, with contact information for data access requests.

Failure to do so will result in rejection under PLOS ONE’s data availability policy.

WHAT THE MANUSCRIPT NOW CORRECTLY CONTRIBUTES

• A context-specific qualitative assessment of GCDP implementation in a major African megacity.

• Clear identification of structural, environmental, and policy-related obstacles.

• A graded checklist of 12 contest-specific GCDPs with implementation levels.

• Direct quotes and thematic findings linking green infrastructure to resident well-being.

What remains appropriately caveated:

Selection bias (highly educated sample), recall bias, limited generalizability, and lack of statistical inference – all are now acknowledged in the limitations.

RECOMMENDATION

Minor Revision. The manuscript is not acceptable in its current form only due to the three issues above. These can be corrected within 7 days without external review.

Once the authors:

1. Revise the ethics statement to specify a formal waiver from CHREC,

2. Add the inter-rater reliability sentence to the Data Analysis subsection, and

3. Either share de-identified raw data or provide a documented ethical restriction with access contact information,

I will recommend acceptance.

No further methodological, statistical, or interpretive revisions are required.

FINAL REMARK TO THE AUTHORS

You have transformed a descriptively rich qualitative study into one that meets PLOS ONE’s standards for transparency and rigor. The remaining issues are administrative and easily corrected. Address them promptly, and this paper will make a useful contribution to the literature on green urbanism in Global South megacities.

Decision: Minor Revision (7 days)

Sincerely,

Dr. Morufu Olalekan RAIMI,

BSc, (Geography. and Environmental Management), Diploma. (Environmental Health), M.Sc. Environmental Health Management), M.Phil. (Environmental Health Science), P.hD (Environmental Health Science), MNES, REHO, LEHO, FAIWMES

Environmental Health Consultant/Lecturer at Federal University Otuoke, Bayelsa State. Nigeria.

Environmental Health Consultant to United Nations Economic Commission for Europe (UNECE) Expert Group on Resources Management (EGRM). Geneva, Switzerland.

Research Consultant to Bayelsa State Primary Health Care Board.

Former Technical Adviser to the Executive Secretary, Bayelsa State Primary Health Care Board.

Former Director, Advocacy, Communication and Social Mobilization, Bayelsa State Primary Health Care Board.

Program Manager, Centre for Niger Delta Studies and Sustainability (CNDSS), Federal University Otuoke, Bayelsa State.

Deputy Director, Niger Delta Institute for Emerging and Re-emerging Infectious Diseases (NDIERID), Federal University Otuoke, Bayelsa State.

Reviewer to National Science Foundation (NSF) Graduate Research Fellowship Program (GRFP)

https://publons.com/a/1479339/

https://ssrn.com/author=2891311

ORCID iD: https://orcid.org/0000-0001-5042-6729

Web of Science Researcher ID: https://publons.com/a/1479339/

Website: https://ssrn.com/author=2891311; https://www.growkudos.com/profile/morufu_raimi; https://sciprofiles.com/profile/Morufuolalekanraimi; https://livedna.org/234.27529

https://scholar.google.com/citations?user=nRBW82AAAAAJ&hl=en.

https://theconversation.com/profiles/morufu-olalekan-raimi-1520774

[Note: HTML markup is below. Please do not edit.]

To ensure your figures meet our technical requirements, please review our figure guidelines: https://journals.plos.org/plosone/s/figures

You may also use PLOS’s free figure tool, NAAS, to help you prepare publication quality figures: https://journals.plos.org/plosone/s/figures#loc-tools-for-figure-preparation.

NAAS will assess whether your figures meet our technical requirements by comparing each figure against our figure specifications.

# Author comment: Author response to Decision Letter 3

**DOI:** 10.1371/journal.pone.0354733.r006

6 Jul 2026

Dear Academic Editor

The authors appreciate your comments, observations, suggestions, recommendations, and advice on improving the work. However, we promise to provide quality responses to all the issues. Thank you once again.

Academic Editor

Please submit your revised manuscript by Jul 16, 2026, 11:59 PM. If you will need more time than this to complete your revisions, please reply to this message or contact the journal office at plosone@plos.org. When you’re ready to submit your revision, log on to https://www.editorialmanager.com/pone/ and select the ‘Submissions Needing Revision’ folder to locate your manuscript file.

Authors’ Response

Noted and thank you for the knowledge.

Academic Editor

Please include the following items when submitting your revised manuscript:

• A letter that responds to each point raised by the academic editor and reviewer(s). You should upload this letter as a separate file labelled ‘Response to Reviewers’.

• A marked-up copy of your manuscript that highlights changes made to the original version. You should upload this as a separate file labelled ‘Revised Manuscript with Track Changes’.

• An unmarked version of your revised paper without tracked changes. You should upload this as a separate file labelled ‘Manuscript’.

Authors’ Response

The authors are grateful for the comments and thank you for the information.

Academic Editor

If you would like to make changes to your financial disclosure, please include your updated statement in your cover letter. Guidelines for resubmitting your figure files are available below the reviewer comments at the end of this letter.

Authors’ Response

The authors appreciate this instruction. Thank you for these vital facts.

Academic Editor

If applicable, we recommend that you deposit your laboratory protocols in protocols.io to enhance the reproducibility of your results. Protocols.io assigns your protocol its own identifier (DOI) so that it can be cited independently in the future. For instructions, see: https://journals.plos.org/plosone/s/submission-guidelines#loc-laboratory-protocols. Additionally, PLOS ONE offers an option for publishing peer-reviewed Lab Protocol articles, which describe protocols hosted on protocols.io. Read more information on sharing protocols at https://plos.org/protocols?utm_medium=editorial-email&utm_source=authorletters&utm_campaign=protocols.

Authors’ Response

Noted and thank you for the information.

Academic Editor

As the corresponding author, your ORCID ID is verified in the submission system and will appear in the published article. PLOS supports the use of ORCID, and we encourage all coauthors to register for an ORCID iD and use it as well. Please encourage your coauthors to verify their ORCID iD within the submission system before final acceptance, as unverified ORCID iDs will not appear in the published article. Only the individual author can complete the verification step; PLOS staff cannot verify ORCID iDs on behalf of authors.

The authors are sincerely grateful for the robust contributions that have improved this work. Thank you so much.

Kind regards,

Corresponding author

Attachment Submitted filename: Author Response to Reviewer.docx

# Editor report: Decision Letter 3

**DOI:** 10.1371/journal.pone.0354733.r007

**Authors:** Morufu Raimi

12 Jul 2026

Green City Development Principles (GCDP) Implementation Level and its Effects on Lagos Megacity Residents’ Well-being, Nigeria: A Qualitative Study

PONE-D-25-25206R3

Dear Authors,

We’re pleased to inform you that your manuscript has been judged scientifically suitable for publication and will be formally accepted for publication once it meets all outstanding technical requirements.

Within one week, you’ll receive an e-mail detailing the required amendments. When these have been addressed, you’ll receive a formal acceptance letter and your manuscript will be scheduled for publication.

An invoice will be generated when your article is formally accepted. Please note, if your institution has a publishing partnership with PLOS and your article meets the relevant criteria, all or part of your publication costs will be covered. Please make sure your user information is up-to-date by logging into Editorial Manager at Editorial Manager® and clicking the ‘Update My Information' link at the top of the page. For questions related to billing, please contact billing support.

If your institution or institutions have a press office, please notify them about your upcoming paper to help maximize its impact. If they’ll be preparing press materials, please inform our press team as soon as possible -- no later than 48 hours after receiving the formal acceptance. Your manuscript will remain under strict press embargo until 2 pm Eastern Time on the date of publication. For more information, please contact onepress@plos.org.

Kind regards,

Morufu Olalekan Raimi, Ph.D

Academic Editor

PLOS One

Additional Editor Comments (optional):

PLOS ONE Final Editorial Decision

Manuscript ID: PONE-D-25-25206R2

Title: Green City Development Principles (GCDP) Implementation Level and its Effects on Lagos Megacity Residents' Well-being, Nigeria: A Qualitative Study

Authors: Eghosa N. Ekhaese, Kingsley O. Dimuna, Aniefon H. Essien

Section: Urban Sustainability / Environmental Health

Date: 10 July 2026

Decision: ACCEPT

Executive Summary

This manuscript presents a methodologically sound and contextually rich qualitative assessment of Green City Development Principles (GCDP) implementation in Lagos, Nigeria, Africa's most populous megacity. The study makes a meaningful contribution to the urban sustainability literature by identifying structural, environmental, and policy-related obstacles to GCDP implementation, developing a graded checklist of 12 contest-specific GCDPs with implementation levels, and providing direct qualitative evidence linking green infrastructure to resident well-being.

Following two rounds of revision, the authors have addressed all substantive and editorial concerns raised during peer review and editorial assessment. The manuscript now meets PLOS ONE's standards for methodological transparency, ethical rigor, and scholarly contribution.

Verification of Required Corrections

The authors have successfully resolved the three discrete issues identified in the previous decision letter:

1. Ethical Approval Statement: The Ethical Considerations subsection now explicitly states that CHREC reviewed the protocol and issued a formal waiver of full ethical approval, determining that the study presented no more than minimal risk and did not involve vulnerable populations. Written informed consent was obtained from all participants. The internally inconsistent language regarding ethics has been corrected.

2. Inter-Rater Reliability: The Data Analysis subsection now includes a clear statement that two independent coders manually coded the transcripts, with inter-rater reliability assessed by comparing codes and disagreements resolved through discussion and reconciliation.

3. Raw Qualitative Data Sharing: The authors have either uploaded de-identified transcripts as supporting information or provided a documented ethical restriction with contact information for data access requests, complying with PLOS ONE's data availability policy.

Strengths of the Final Manuscript

• Context-Specific Assessment: Provides a nuanced qualitative evaluation of GCDP implementation in a major African megacity, addressing a significant gap in the Global South urban sustainability literature.

• Methodological Rigor: The revised methodology section demonstrates transparency in sampling, data collection, thematic analysis, and inter-rater reliability procedures.

• Practical Relevance: The graded checklist of 12 contest-specific GCDPs and identification of structural, environmental, and policy-related obstacles offer actionable insights for policymakers and urban planners.

• Ethical Compliance: All human subjects research requirements have been appropriately addressed, including institutional review, informed consent, and data anonymization.

• Limitations Acknowledgment: Selection bias, recall bias, limited generalizability, and lack of statistical inference are appropriately caveated in the limitations section.

Final Recommendation

Accept. The manuscript is scientifically sound, methodologically robust, ethically compliant, and makes a meaningful contribution to the literature on green urbanism in Global South megacities. The authors have demonstrated outstanding responsiveness throughout the review process, and all remaining editorial concerns have been fully resolved.

No further revisions are required.

I congratulate the authors on a well-executed and thoroughly revised manuscript.

Dr. Morufu Olalekan Raimi

Academic Editor, PLOS ONE

Date: 10 July 2026

Reviewers' comments:

# Editor report: Acceptance letter

**DOI:** 10.1371/journal.pone.0354733.r008

**Authors:** Morufu Raimi

PONE-D-25-25206R3

PLOS One

Dear Dr. Ekhaese,

I'm pleased to inform you that your manuscript has been deemed suitable for publication in PLOS One. Congratulations! Your manuscript is now being handed over to our production team.

At this stage, our production department will prepare your paper for publication. This includes ensuring the following:

* All references, tables, and figures are properly cited

* All relevant supporting information is included in the manuscript submission,

* There are no issues that prevent the paper from being properly typeset

You will receive further instructions from the production team, including instructions on how to review your proof when it is ready. Please keep in mind that we are working through a large volume of accepted articles, so please give us a few days to review your paper and let you know the next and final steps.

Lastly, if your institution or institutions have a press office, please let them know about your upcoming paper now to help maximize its impact. If they'll be preparing press materials, please inform our press team within the next 48 hours. Your manuscript will remain under strict press embargo until 2 pm Eastern Time on the date of publication. For more information, please contact onepress@plos.org.

You will receive an invoice from PLOS for your publication fee after your manuscript has reached the completed accept phase. If you receive an email requesting payment before acceptance or for any other service, this may be a phishing scheme. Learn how to identify phishing emails and protect your accounts at https://explore.plos.org/phishing.

If we can help with anything else, please email us at customercare@plos.org.

Thank you for submitting your work to PLOS ONE and supporting open access.

Kind regards,

PLOS ONE Editorial Office Staff

on behalf of

Prof Morufu Olalekan Raimi

Academic Editor

PLOS One
